# Integrative Analysis Reveals Relationships of Genetic and Epigenetic Alterations in Osteosarcoma

**DOI:** 10.1371/journal.pone.0048262

**Published:** 2012-11-07

**Authors:** Stine H. Kresse, Halfdan Rydbeck, Magne Skårn, Heidi M. Namløs, Ana H. Barragan-Polania, Anne-Marie Cleton-Jansen, Massimo Serra, Knut Liestøl, Pancras C. W. Hogendoorn, Eivind Hovig, Ola Myklebost, Leonardo A. Meza-Zepeda

**Affiliations:** 1 Department of Tumour Biology, The Norwegian Radium Hospital, Oslo University Hospital, Oslo, Norway; 2 Department of Informatics, University of Oslo, Oslo, Norway; 3 Norwegian Microarray Consortium, Department of Molecular Biosciences, University of Oslo, Oslo, Norway; 4 Department of Pathology, Leiden University Medical Center, Leiden, The Netherlands; 5 Laboratory of Experimental Oncology, Istituto Ortopedico Rizzoli, Bologna, Italy; The Chinese University of Hong Kong, Hong Kong

## Abstract

**Background:**

Osteosarcomas are the most common non-haematological primary malignant tumours of bone, and all conventional osteosarcomas are high-grade tumours showing complex genomic aberrations. We have integrated genome-wide genetic and epigenetic profiles from the EuroBoNeT panel of 19 human osteosarcoma cell lines based on microarray technologies.

**Principal Findings:**

The cell lines showed complex patterns of DNA copy number changes, where genomic copy number gains were significantly associated with gene-rich regions and losses with gene-poor regions. By integrating the datasets, 350 genes were identified as having two types of aberrations (gain/over-expression, hypo-methylation/over-expression, loss/under-expression or hyper-methylation/under-expression) using a recurrence threshold of 6/19 (>30%) cell lines. The genes showed in general alterations in either DNA copy number or DNA methylation, both within individual samples and across the sample panel. These 350 genes are involved in embryonic skeletal system development and morphogenesis, as well as remodelling of extracellular matrix. The aberrations of three selected genes, *CXCL5*, *DLX5* and *RUNX2*, were validated in five cell lines and five tumour samples using PCR techniques. Several genes were hyper-methylated and under-expressed compared to normal osteoblasts, and expression could be reactivated by demethylation using 5-Aza-2′-deoxycytidine treatment for four genes tested; *AKAP12, CXCL5*, *EFEMP1* and *IL11RA*. Globally, there was as expected a significant positive association between gain and over-expression, loss and under-expression as well as hyper-methylation and under-expression, but gain was also associated with hyper-methylation and under-expression, suggesting that hyper-methylation may oppose the effects of increased copy number for detrimental genes.

**Conclusions:**

Integrative analysis of genome-wide genetic and epigenetic alterations identified dependencies and relationships between DNA copy number, DNA methylation and mRNA expression in osteosarcomas, contributing to better understanding of osteosarcoma biology.

## Introduction

Osteosarcoma is the most common non-haematological primary malignant tumour of bone, occurring most commonly in the metaphyseal regions of long bones in adolescents and young adults, but also in patients over 40 years of age [1]. Almost all conventional osteosarcomas are high-grade malignant tumours with poor prognosis, and 20–25% of the patients have detectable metastases at diagnosis [2], [3]. The 5-year survival rate for patients diagnosed with osteosarcoma without presence of metastasis is 60–65% [3], [4], [5], whereas it is only 20–28% for osteosarcoma patients with metastases at diagnosis [3], [6], [7]. Even though the survival rate has improved considerably after the introduction of neoadjuvant chemotherapy, the need for advances in treatment regimens is still high.

Most conventional osteosarcomas have complex karyotypes with numerous and highly variable genomic aberrations. A vast number of DNA copy number changes have been identified using chromosome- and microarray-based comparative genomic hybridisation (CGH and array CGH), more recently also utilizing high-density single nucleotide polymorphism (SNP) microarrays [8], [9], [10]. Few, if any, consistent chromosomal aberrations have been recognized in osteosarcoma, mainly consisting of recurrent alterations in 6p, 8q, 13q and 17p [9], [10], [11], [12]. Many genes become deregulated due to genomic aberrations, and DNA copy number and gene expression data have been combined to identify oncogenes and tumour suppressor genes in osteosarcomas [10], [11], [13], [14]. Another important mechanism for down-regulation of gene expression is DNA methylation, more specifically at CpG sites in the promoter region of genes. It has been speculated that epigenetic mechanisms may be more prevalent than mutation in childhood cancers like retinoblastoma [15]. Although a number of research groups have reported comparisons of alterations in DNA copy number, DNA methylation and mRNA expression for other types of cancers [16], [17], [18], only a few studies have examined the interdependence of these types of mechanisms in osteosarcoma [19], [20]. The benefits of an integrative approach are that driver genes and their regulatory mechanisms may be identified, as well as relationships between mechanisms. The identification of molecular markers and pathways contributing to osteosarcoma development and progression may facilitate better diagnosis and prognostication, as well as the development of new treatment strategies.

As part of EuroBoNeT, a European Network of Excellence on bone tumours (http://www.eurobonet.eu), we have access to a large collection of clinical samples and resources for pre-clinical studies. One such resource is a collection of 19 osteosarcoma cell lines, which have been previously characterised in detail, including DNA fingerprinting to guarantee their identity [21]. Genetic, phenotypic and functional characterisation have shown that these cell lines robustly represent osteosarcoma clinical samples [21], [22], [23]. The EuroBoNeT osteosarcoma cell line panel will serve as a highly valuable, well-characterised model system for basic and pre-clinical studies.

By using various microarray technologies, genome-wide genetic and epigenetic changes were analysed in the EuroBoNeT osteosarcoma cell line panel. DNA copy number changes have been mapped at high resolution using the Affymetrix Genome-Wide Human SNP Array 6.0, DNA methylation status of approximately 27,000 CpG sites have been identified using the Illumina HumanMethylation27 BeadChip and global mRNA expression data have been obtained using the Illumina HumanWG-6 v2 Expression BeadChip. The different levels of genome-wide information have been analysed separately and integrated in order to identify recurrently altered genes showing more than one type of aberration, as well as the dependencies of the different types of aberrations in osteosarcomas.

## Results

### Genetic and Epigenetic Alterations in Osteosarcoma Cell Lines

DNA copy number changes in the EuroBoNeT panel of 19 human osteosarcoma cell lines [21] were mapped at high resolution using the Affymetrix Genome-Wide Human SNP Array 6.0, DNA methylation status of approximately 27,000 CpG sites was identified using the Illumina HumanMethylation27 BeadChip and global mRNA expression data were obtained using the Illumina HumanWG-6 v2 Expression BeadChip. For the two latter types of data, two normal osteoblast and four normal bone samples were included as controls. Clinical data for the osteosarcoma cell lines and normal samples are given in Table S1.

**Figure 1:**
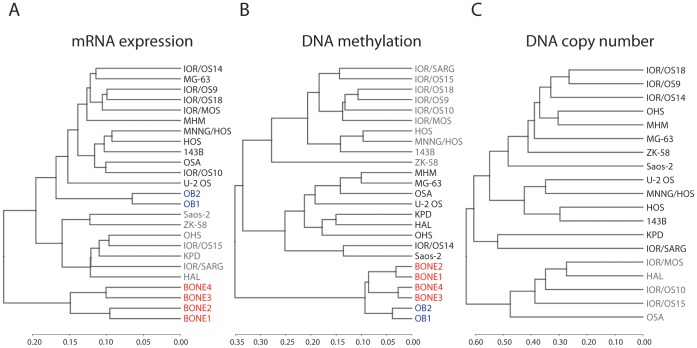
Hierarchical clustering of osteosarcoma cell lines and normal samples. Dendrograms from unsupervised hierarchical clustering of the osteosarcoma cell lines, normal bone and normal osteoblast samples based on genome-wide (A) mRNA expression (vst transformed and quantile normalised probe intensities), (B) DNA methylation (probe beta values) and (C) DNA copy number (probe intensities). The osteosarcoma cell lines have been colour-coded in gray and black, highlighting the two main subclusters, the normal bone samples in red and the normal osteoblast samples in blue. The clusters were made using Spearman correlation as distance measure and complete linkage.

Unsupervised hierarchical clustering of the 19 cell lines and 6 normal samples was performed in R v.2.13.0 using the three types of microarray data, and the resulting cluster dendrograms are shown in Figure 1. The clustering was performed using the genome-wide probe intensities for the DNA copy number data, avgBeta (average ratio of signal from probe detecting methylation relative to the sum of both probes) probe values for the DNA methylation data and variance-stabilizing transformation (vst) and quantile normalised probe intensities for the mRNA expression data. Based on the distance of the dendrograms, the cell lines appeared more similar based on overall gene expression than copy number, with methylation in between. The cell lines clustered in general differently based on each type of data, although some similarities were seen, such as the co-clustering of IOR/OS9 and IOR/OS18 for all data types. The HOS cell line and its derivatives 143B and MNNG/HOS clustered together for all data types, with HOS and MNNG/HOS being more similar in terms of gene expression and methylation, and 143B and HOS in terms of copy number. The clustering patterns did not correlate with the clinical information associated with the sample of origin (Table S1), the cell line phenotypes, including the status of *CDKN2A*, *MDM2* and *TP53*, nor with the differentiation capacity or *in vivo* tumour formation capacity [21], [22].

Furthermore, all the normal samples clustered together in one branch based on the methylation data, whereas the osteoblasts clustered together with the osteosarcoma cell lines for the expression data. Based on the distance of the dendrograms, the normal samples were more similar to each other than the osteosarcoma cell lines were, especially for the methylation data. Since the clustering pattern of osteoblasts and bone samples was markedly different for the expression data, the further comparisons of methylation and expression levels in the osteosarcoma cell lines were performed against only the osteoblasts. The osteosarcoma cell lines and osteoblasts are both *in vitro* grown samples, and would be expected to better separate cancer-associated properties.

**Figure 2:**
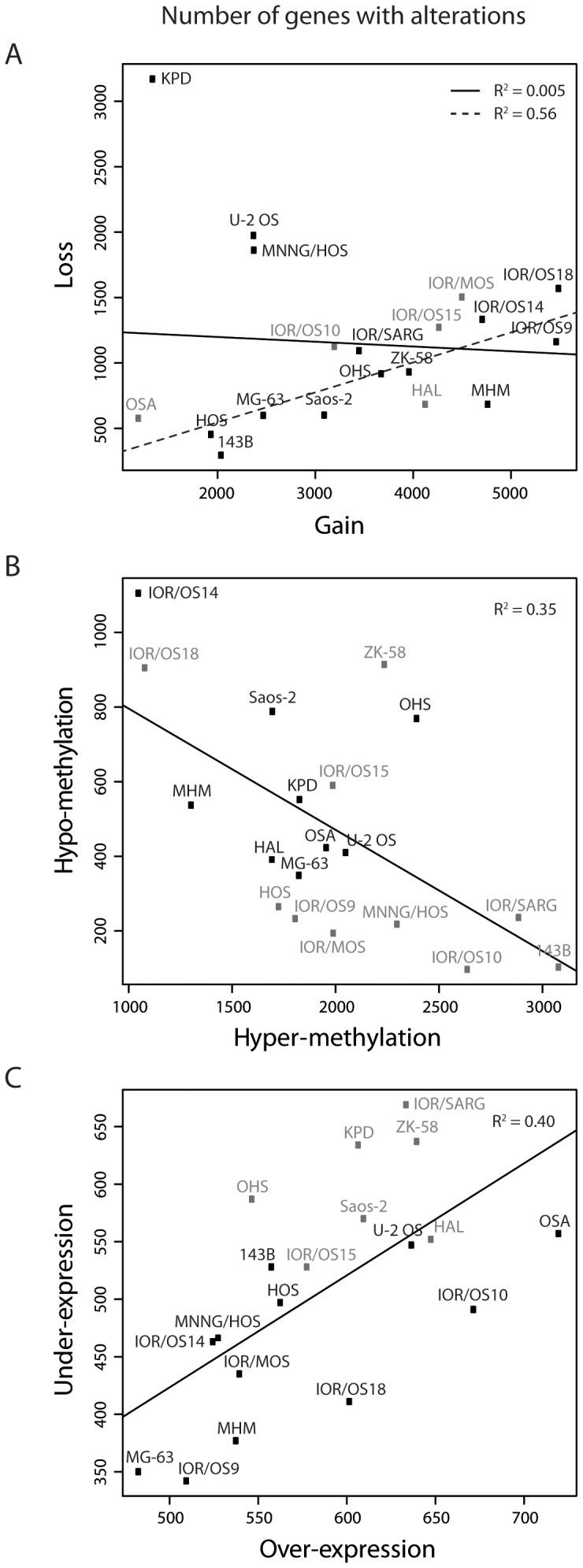
Number of genes with alterations. Plot of the number of genes with (A) gain and loss, (B) hyper- and hypo-methylation and (C) over- and under-expression for all the cell lines. The linear regression line is indicated in black. For the copy number (A), the linear regression line omitting the outlier samples U-2 OS, MNNG/HOS and KPD is indicated with a dashed line. The cell lines are colour-coded in gray and black according to the separation into two main subclusters from the respective unsupervised hierarchical clustering (Figure 1).

For each cell line, genes with DNA copy number aberrations (gain and loss) were identified using the SNP rank segmentation algorithm in Nexus. Probes detecting variation in DNA methylation (hyper- and hypo-methylation) compared to the normal osteoblasts were identified using a cut-off of deltaBeta >0.4 and < −0.4, whereas probes detecting variation in mRNA expression (over- and under-expression) compared to the normal osteoblasts were identified using a cut-off of vst transformed and quantile normalised ratio >1 and < −1. The probes were collapsed to gene level for the analyses, keeping the probe level information. The number of genes with each type of aberration for all the cell lines are plotted in Figure 2 and listed in Table S2.

For the copy number changes, most cell lines showed more genes with gains than with losses (Figure 2A). The cell lines U-2 OS and MNNG/HOS had a different pattern, with almost similar numbers of genes gained and lost, whereas KPD diverged from all the other cell lines having a higher number of genes lost than gained. Excluding these three outliers, there was a correlation between the number of genes with gain or loss (R^2^ = 0.56). The distribution of number of genes gained and lost did not correlate with the clustering pattern based on the copy number changes (Figure 1).

As expected, most cell lines showed more hyper-methylation than hypo-methylation, and there was an inverse correlation between the number of genes hyper- and hypo-methylated (Figure 2B, R ^2^ = 0.35). The cell line 143B had almost 20 times more genes hyper-methylated than hypo-methylated, whereas IOR/OS14 had slightly more genes hypo-methylated than hyper-methylated. The distribution of number of genes hyper- and hypo-methylated showed a trend to correlate with the clustering pattern (Figure 1). The two main subclusters showed different distributions with respect to the number of genes hyper- and hypo-methylated, with the exception of the cell lines IOR/OS15 and IOR/OS18. The DNA copy number, DNA methylation and mRNA expression levels of the methyltransferase genes DNA (cytosine-5-)-methyltransferase 1, -3A and -3B (*DNMT1*, *-3A* and *-3B*) are shown in Figure S1. The genes were gained in several cell lines and hyper-methylated in some, but all cell lines except HAL showed similar mRNA expression levels as the normal osteoblasts. No correlations were found between the DNA copy number, DNA methylation and mRNA expression levels of *DNMT1, -3A* and *-3B*, and the number of hyper- and hypo-methylated genes.

The number of genes over- and under-expressed was more even, and there was a correlation between the number of genes over- and under-expressed (Figure 2C, R ^2^ = 0.40). The distribution of the number of over- and under-expressed genes reflected also partly the clustering pattern (Figure 1). The cell lines in one of the two main subclusters showed in general higher numbers of under-expressed genes.

A genome-wide frequency plot of alterations in DNA copy number is given in Figure S2. The cell lines showed more gains than losses, and an increased copy number of regions in almost every chromosome was present in more than 50% of the samples. The most frequent gains were regions in 2p, 14q, 20q and 8q, whereas the most frequent losses were regions in 13q, 3p and 6q. A genome-wide analysis using The Genomic HyperBrowser (http://hyperbrowser.uio.no/hb/) [24] identified an over-representation of gene-rich areas among frequently gained regions and gene-poor areas among frequently lost regions. The analysis was performed as two separate Monte Carlo-based hypothesis tests, for gain and loss respectively, giving p-value <0.001 in both cases. Tests were also performed separately for each chromosome arm (except the sex chromosomes), resulting in 27/39 significant arms for gain and all 39 arms significant for loss. Figure S3 shows the frequency plot of copy number aberrations and gene density for chromosome arms 2q, 8p, 19p and 19q, all with significant results for both gain and loss tests. The chromosome arms that were not significant for gain were 3p, 4p, 4q, 6q, 10p, 11p, 12p, 13q, 14q, 17q, 18p and 18q, and the frequency plot of copy number aberrations and gene density for these chromosome arms is shown in Figure S4.

The methylation data were analysed with the Bioconductor packages Limma and MethyLumi to identify differentially methylated genes compared to the normal osteoblasts. Using a cut-off of M-value (log_2_ ratio of intensity of probes detecting methylation and no methylation) >6, 328 significantly differentially methylated genes were identified, listed in Table S3. The gene list was analysed for functional enrichment in DAVID (Database for Annotation, Visualization and Integrated Discovery), and the top five terms in the top three clusters are listed in Table 1. The first cluster contained terms involving embryonic organ development and morphogenesis, as well as homeobox proteins and DNA binding, the second cluster contained terms involving thyroglobulin, whereas the third cluster contained terms involving potassium channel and ion transport. The top 10 clusters with all terms are listed in Table S4.

**Table 1:** Enrichment analysis of differentially methylated genes using DAVID.

Cluster number	Enrichment score	Term	Counts	Population hits	FDR
1	5.13	Embryonic morphogenesis	25	307	4.7E-06
		Sequence-specific DNA binding	35	607	1.2E-05
		DNA-binding region:Homeobox	17	190	1.1E-04
		Embryonic organ development	17	172	2.2E-04
		Chordate embryonic development	22	331	1.6E-03
2*	3.47	Thyroglobulin type-1	5	17	0.24
		TY	5	17	0.27
		Thyroglobulin type-1	4	13	1.58
3	3.34	Voltage-dependent potassium channel	8	33	1.6E-03
		Ion transport	27	578	2.5E-03
		Potassium channel	10	78	5.3E-03
		Voltage-gated channel	12	150	0.04
		Potassium voltage-gated channel, alpha subunit, subfamilies A/C/D/F/G/S	6	20	0.03

The first five terms in the first three clusters are shown, with enrichment score. The counts and population hits are the number of genes in the gene list and background gene list, respectively, mapping to a specific term. FDR, false discovery rate.

* This cluster contained only three terms.

The expression data were analysed with the Bioconductor packages Limma and Lumi to identify differentially expressed genes compared to the normal osteoblasts. Using a cut-off of vst ratio >0.5, 283 significantly differentially expressed genes were identified, listed in Table S5. The gene list was analysed for functional enrichment in DAVID, and the top five terms in the top three clusters are listed in Table 2. The first cluster contained terms involving ribosome and translation, the second cluster terms involving fibrinogen, whereas the third cluster contained terms involving embryonic skeletal system and organ development and morphogenesis, as well as homeobox proteins and DNA binding. The top 10 clusters with all terms are listed in Table S6.

**Table 2:** Enrichment analysis of differentially expressed genes using DAVID.

Cluster number	Enrichment score	Term	Counts	Population hits	FDR
1	3.28	Translational elongation	10	101	0.01
		Ribosome	10	87	0.01
		Ribosome	8	73	0.04
		Cytosolic ribosome	8	81	0.10
		Ribosomal protein	11	188	0.13
2	2.38	Fibrinogen, alpha/beta/gamma chain, C-terminal globular, subdomain 2	3	4	1.35
		Fibrinogen, alpha/beta/gamma chain, C-terminal globular, subdomain 1	4	23	4.19
		Fibrinogen C-terminal	4	32	9.72
		Fibrinogen, alpha/beta/gamma chain, C-terminal globular	4	32	10.5
		FBG	4	32	9.21
3	2.28	Embryonic skeletal system development	9	77	0.01
		Embryonic skeletal system morphogenesis	8	57	0.01
		Embryonic organ morphogenesis	10	133	0.09
		Skeletal system development	15	319	0.11
		Embryonic organ development	11	172	0.13

The first five terms in the first three clusters are shown, with enrichment score. The counts and population hits are the number of genes in the gene list and background gene list, respectively, mapping to a specific term. FDR, false discovery rate.

### Identification of Recurrently Altered Genes Using Genetic and Epigenetic Information

The lists of genes with alterations in DNA copy number, DNA methylation or mRNA expression level were combined for each cell line in order to identify genes showing more than one type of aberration. The 11,843 genes from chromosome 1–22 common to the three microarray platforms were included in the analyses. With two types of changes for each of the three data sets (gain and loss, hyper- and hypo-methylation, over- and under-expression), 12 two-way and 8 three-way combinations are possible. The combined lists with genes showing more than one type of aberration for the individual cell lines were subsequently compared in order to identify recurrently altered genes.

**Figure 3:**
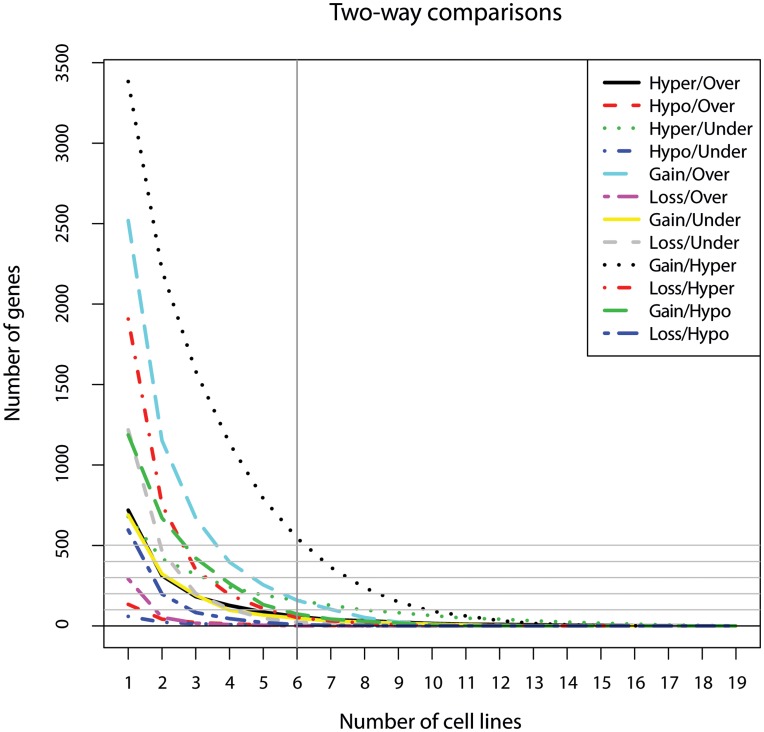
Number of genes with alterations for two-way combinations. Plot of the number of genes with alterations for all 12 two-way combinations at different sample recurrence thresholds. The recurrence threshold of 6/19 cell lines (>30%) is indicated with a black line.

The number of genes showing more than one type of aberration is plotted as a function of number of samples with this occurrence for all two-way combinations in Figure 3. As can be seen, the combination gain and hyper-methylation had the highest frequency of occurrence, followed by gain and over-expression. In 6/19 cell lines (>30%), 546 genes were both gained and hyper-methylated, followed by 159 genes showing both gain and over-expression and 158 genes showing both hyper-methylation and under-expression.

The number of genes showing more than one type of aberration is plotted as a function of number of samples with this occurrence for all three-way combinations in Figure S5. In this case, 16 genes were gained, hyper-methylated and under-expressed in 6/19 cell lines (>30%), followed by 12 genes showing gain, hyper-methylation and over-expression. A recurrence plot for each data type is also given in Figure S5, showing that most genes with recurrent alterations were gained, followed by hyper-methylation.

To identify genes with altered expression level correlating with aberrations in DNA copy number or DNA methylation, the four two-way combinations gain/over-expression, hypo-methylation/over-expression, loss/under-expression and hyper-methylation/under-expression were considered. Using the sample recurrence threshold of six or more cell lines (>30%), these four combinations made up a total of 335 genes. The combinations gain/over-expression and hyper-methylation/under-expression gave the highest number of genes, 159 and 158, respectively. Of the 335 genes, only 11 showed simultaneous aberrations in both DNA copy number and DNA methylation. For genes with multiple probes, usually the same probes showed recurrent alterations.

Since changes in DNA copy number and DNA methylation may be alternative mechanisms for altering mRNA expression levels in the same direction, it was also investigated if genes with recurrent over-expression were either gained or hypo-methylated and if genes with recurrent under-expression were either lost or hyper-methylated in a total of six or more cell lines. However, only 15 additional genes were identified in this way, giving a total number of 350 recurrently altered genes. Thus, the majority of these genes showed alterations in either DNA copy number or DNA methylation, both within individual samples and across the sample panel.

This list of 350 genes, annotated with type of deviation and recurrence count, is given in Table S7.

**Figure 4:**
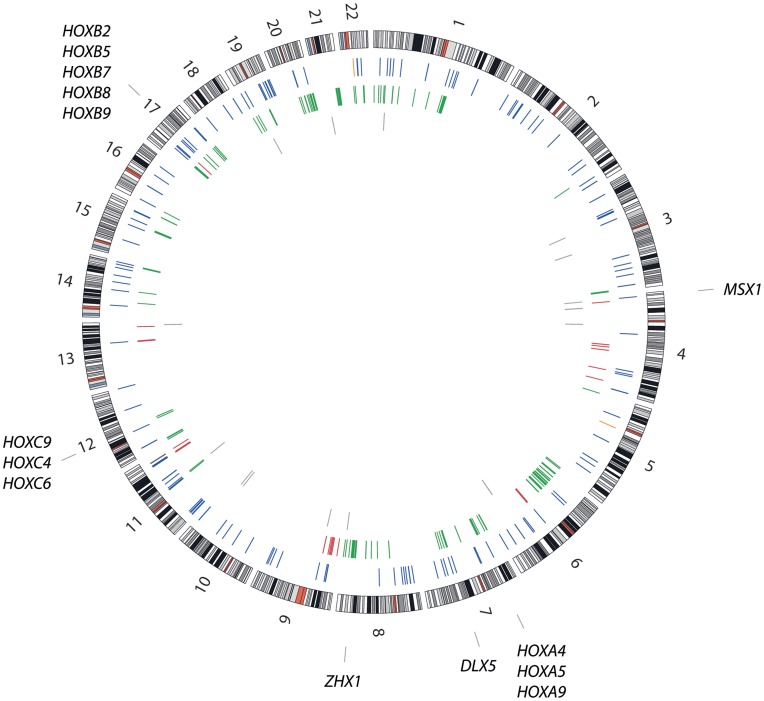
Genomic location of 350 genes that recurrently showed two types of aberrations. Circos plot showing the genomic location of the 350 genes that recurrently showed two types of aberrations. The locations are colour-coded according to the type of aberrations; orange, hypo-methylation/over-expression; blue, hyper-methylation/under-expression; green, gain/over-expression; red, loss/under-expression; gray, (gain or hypo-methylation)/over-expression and (loss or hyper-methylation)/under-expression. The genomic location of the 14 homeobox genes in the list is indicated in the outermost circle.

The genomic locations of these 350 genes are visualised using Circos v0.52 in Figure 4. The genes were distributed rather evenly over all chromosomes, but clusters of hyper-methylated and under-expressed genes were present in 3p, 11p and 19q. Clusters of gained and over-expressed genes were also present in 1q, 6p, 8q, 20q and 21q, whereas a cluster of lost and under-expressed genes was present in 9p. The homeobox genes were grouped in three gene clusters, located in 7p, 12q and 17q.

The gene list contained, among others, well-known oncogenes like cyclin-dependent kinase 4 (*CDK4*) and v-myc myelocytomatosis viral oncogene homolog (avian) (*MYC*), both gained and over-expressed, as well as transcription factors involved in normal bone development, like runt-related transcription factor 2 (*RUNX2*) and twist homolog 1 (Drosophila) (*TWIST1*). *RUNX2* was frequently gained and over-expressed, whereas *TWIST1* was frequently hyper-methylated and under-expressed in the cell lines compared to the osteoblasts. The list also contained a number of homeobox genes, 11 HOX family genes (*HOXA4*, -*A5*, *-A9*, *-B2*, *-B5*, *-B7*, *-B8*, *-B9*, *-C4*, *-C6* and *-C9*) and three other genes; distal-less homeobox 5 (*DLX5*), msh homeobox 1 (*MSX1*) and zinc fingers and homeoboxes 1 (*ZHX1*). All homeobox genes were frequently gained and over-expressed, except *MSX1* that was hyper-methylated and under-expressed.

**Figure 5:**
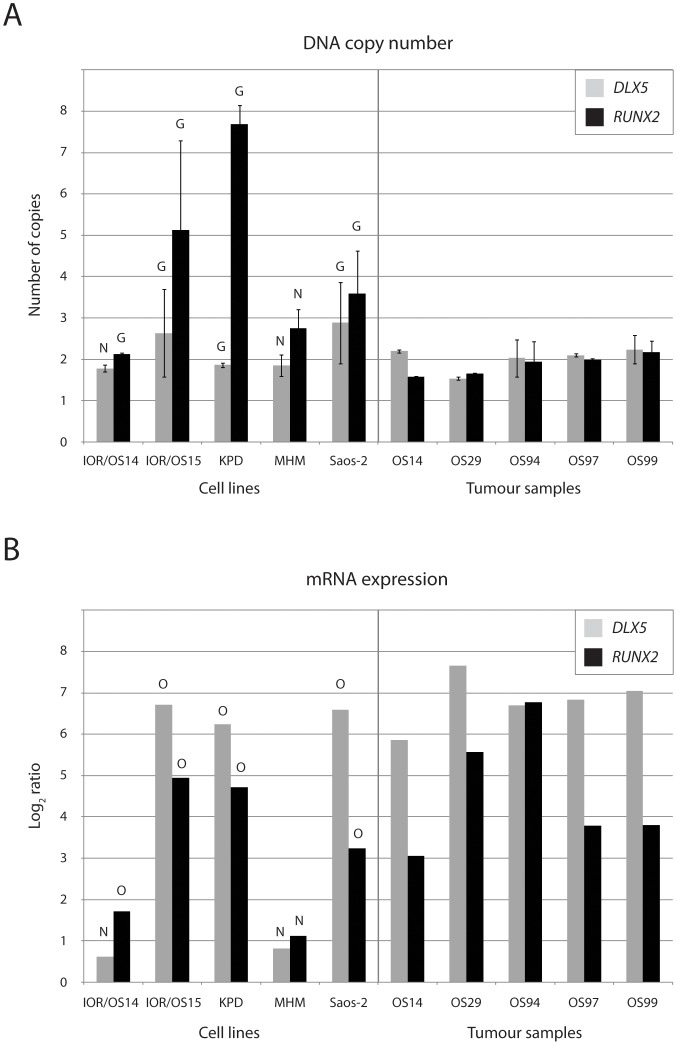
DNA copy number and mRNA expression of *DLX5* and *RUNX2.* Plot of (A) DNA copy number levels of *DLX5* and *RUNX2* based on quantitative real-time PCR and (B) mRNA expression levels of *DLX5* and *RUNX2* based on quantitative real-time RT-PCR, in five cell lines and five tumour samples. The DNA copy number levels have been normalised to the average copy number of two control genes, *EEF1G* and *FBXO11*, whereas the mRNA expression levels have been normalised to the expression of the house-keeping gene *GAPDH* and then to the average expression level of the two normal osteoblast samples. The DNA copy number and mRNA expression levels based on the microarray data are indicated for the cell lines; N, normal copy number/expression; G, gain; O, over-expression.

*DLX5* and *RUNX2* were gained and over-expressed in 6 and 7 of the 19 cell lines, respectively. The aberrations of these genes were validated in five of the cell lines and five osteosarcoma tumour samples using quantitative real-time PCR and RT-PCR, respectively. Clinical data for the tumour samples are given in Table S1. Figure 5 shows the DNA copy number and mRNA expression levels of *DLX5* and *RUNX2*. For the cell lines, the PCR and microarray data correlated well, except for the DNA copy number of *RUNX2* in IOR/OS14 and *DLX5* in KPD, where the PCR data showed normal copy number and not gain. All the tumour samples showed normal copy number of the genes, but showed increased expression of both *DLX5* and *RUNX2*, at similar levels as the cell lines showing over-expression.

The gene list was analysed for functional enrichment in DAVID, and the top five terms in the top three clusters are listed in Table 3. The first and third clusters both contained terms involving extracellular matrix, and terms involving signal peptide and collagen, respectively, whereas the second cluster contained terms involving embryonic skeletal system development and morphogenesis, as well as homeobox protein. The top 10 clusters with all terms are listed in Table S8.

**Table 3:** Enrichment analysis of 350 genes that recurrently showed two types of aberrations using DAVID.

Cluster number	Enrichment score	Term	Counts	Population hits	FDR
1	4.14	Extracellular matrix	28	269	1.5E-04
		Secreted	66	1247	0.005
		Signal	105	2333	0.005
		Signal peptide	105	2333	0.006
		Extracellular region part	48	811	0.047
2	4.08	Skeletal system development	34	281	1.2E-07
		Embryonic morphogenesis	27	255	2.6E-04
		Embryonic skeletal system development	14	69	3.7E-04
		Short sequence motif:Antp-type hexapeptide	8	22	0.004
		Homeobox protein, antennapedia type, conserved site	8	23	0.005
3	3.45	Extracellular matrix	28	269	1.5E-04
		Trimer	9	23	2.6E-04
		Extracellular matrix	21	192	0.001
		Proteinaceous extracellular matrix	25	247	0.001
		Collagen	9	31	0.005

The first five terms in the first three clusters are shown, with enrichment score. The counts and population hits are the number of genes in the gene list and background gene list, respectively, mapping to a specific term. FDR, false discovery rate.

**Figure 6:**
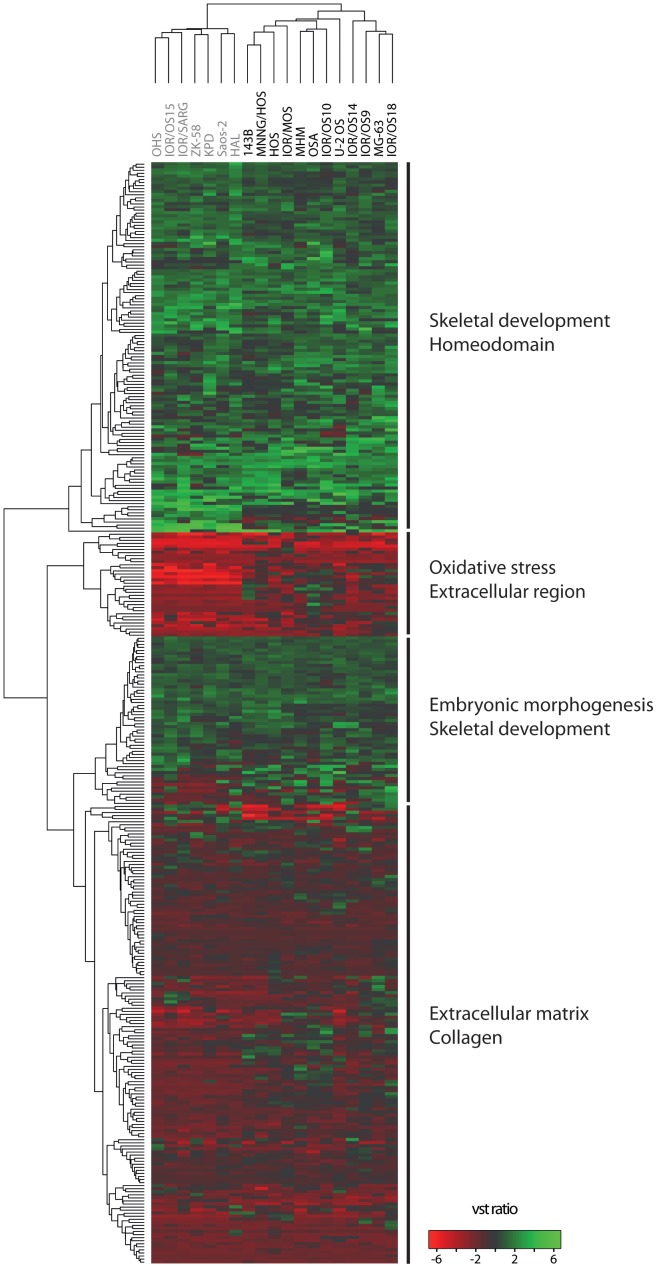
Hierarchical clustering based on 350 genes that recurrently showed two types of aberrations. Hierarchical clustering of the osteosarcoma cell lines based on the expression level of the 350 genes that recurrently showed two types of aberrations. The main terms from functional enrichment analysis using DAVID is indicated for each main subcluster of genes. The cell lines are colour-coded in gray and black according to the separation into two main subclusters from the unsupervised hierarchical clustering based on the global mRNA expression (Figure 1A). The cluster was made using Euclidian as distance measure and complete linkage. Green, increased gene expression; red, decreased gene expression.

Hierarchical clustering of the cell lines based on the expression level of these 350 genes is shown in Figure 6. Again, the clustering patterns correlated neither with the clinical information (Table S1) nor the known properties of the cell lines [21], [22], but the cell lines separated in two main subgroups identical to the cluster based on the global gene expression data. The main terms from functional enrichment analysis using DAVID is indicated for each main subcluster of genes, showing mainly over-expression of genes associated with the terms skeletal development and homeodomain, whereas genes associated with the terms extracellular matrix, oxidative stress and collagen were mainly under-expressed. The same figure with all gene names shown is given in Figure S6.

A functional enrichment analysis was also performed for the 159 genes with gain and over-expression separately, generating a first cluster with terms involving embryonic skeletal system development and homeodomain. The top 10 clusters with all terms are listed in Table S9. A similar analysis of the 158 genes with hyper-methylation and under-expression generated a first cluster with the term extracellular matrix organisation. The top 10 clusters with all terms are listed in Table S10. The other two-way combinations hypo-methylation/over-expression and loss/under-expression did not generate any significant functional terms due to the low number of altered genes.

### Relationships between Different Mechanisms for Alteration of Gene Regulation

**Figure 7:**
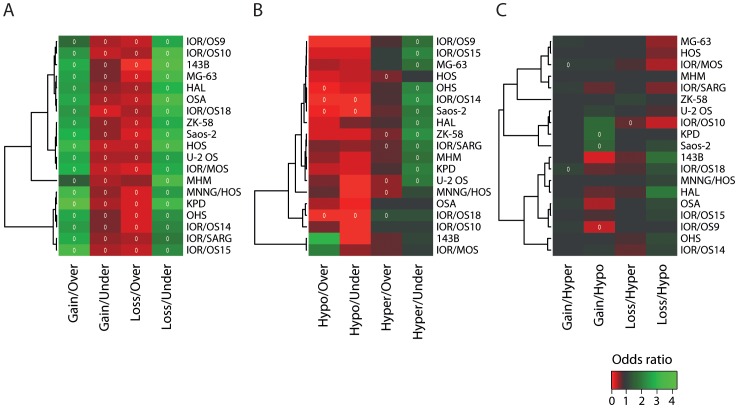
Data dependencies for two-way combinations. Heat map plots visualising the odds ratio and significance of data dependencies, with unsupervised hierarchical clustering of the cell lines, for two-way combinations of (A) DNA copy number and gene expression, (B) DNA methylation and mRNA expression and (C) DNA copy number and DNA methylation. The colours of the heat map plot represent the odds ratio for a gene of having one type of aberration given that it has another type of aberration. Green, positive association (odds ratio >1); black, no association (odds ratio = 1) and red, negative association (odds ratio <1). A white circle indicates significance (Benjamini & Hochberg-corrected chi-square p-value <0.05).

The effects of alterations in DNA copy number and DNA methylation on mRNA expression were examined globally, as well as how the different types of aberrations (gain and loss, hyper- and hypo-methylation, over- and under-expression) related to each other. Heat maps visualising the odds ratios and significance of data dependencies for the 12 two-way combinations are shown in Figure 7. The odds ratio is a measure of effect size, describing the strength of association or non-independence between two binary data values. The significance was determined using Bonferroni-corrected chi-square p-values (p-value <0.05). The cell lines were clustered based on the odds ratios for the different categories of two-way combinations. No clear pattern between the clustering for the different categories was identified. Here, the related cell lines HOS, 143B and MNNG/HOS did not cluster together. Again, the clustering patterns correlated neither with the clinical information (Table S1) nor the known properties of the cell lines [21], [22].

The dependencies between DNA copy number and mRNA expression were comparatively strong and significant for all cell lines. There was a positive association of gain/over-expression and loss/under-expression, and conversely gain/under-expression and loss/over-expression showed a negative association. For the DNA copy number and DNA methylation, there was in general either no association or a negative association for the different combinations, and only a few cell lines showed significant dependencies of some of the combinations.

The dependencies of DNA methylation and mRNA expression were significant in some of the cell lines, particularly for the combination hyper-methylation and under-expression that showed a positive association. The three other two-way combinations had in general a negative association. The two cell lines 143B and IOR/MOS differed from the other cell lines by having a positive association between hypo-methylation and over-expression.

**Figure 8:**
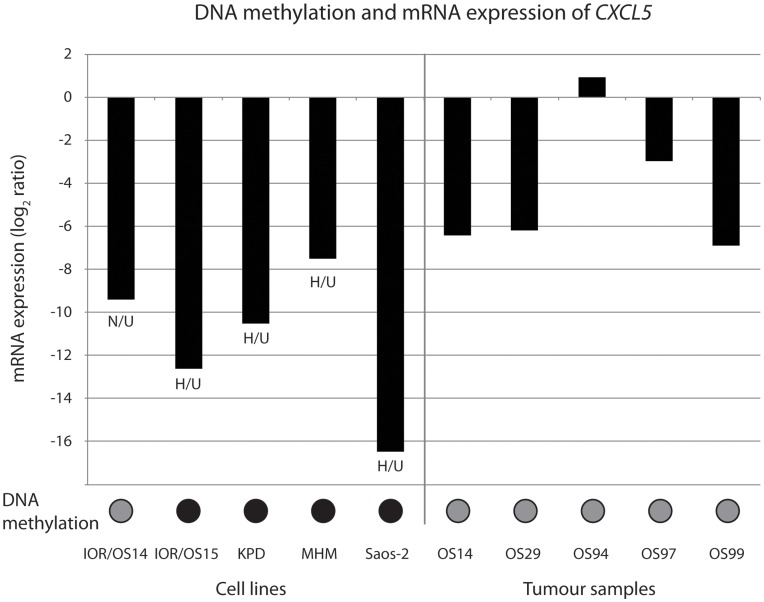
DNA methylation and mRNA expression of *CXCL5.* Plot of the DNA methylation status and mRNA expression level of *CXCL5* based on methylation-specific PCR and quantitative real-time RT-PCR, respectively, in five cell lines and five tumour samples. The mRNA expression levels have been normalised to the expression of the house-keeping gene *GAPDH* and then to the average expression level of the two normal osteoblast samples. The DNA methylation status is indicated with coloured circles; black, full methylation, grey, partial methylation. The DNA methylation and mRNA expression levels based on the microarray data are indicated for the cell lines; N, normal methylation; H, hyper-methylation; U, under-expression.

Methylation of CpG islands in promoter regions may silence gene expression, and is one mechanism for inactivating genes. Of the 350 genes that showed two types of aberrations in at least 6/19 cell lines, 158 genes were hyper-methylated and under-expressed. One of the most frequently hyper-methylated and under-expressed genes was chemokine (C-X-C motif) ligand 5 (*CXCL5*), altered in 18/19 cell lines. Methylation-specific PCR and quantitative real-time RT-PCR were used to validate the promoter methylation status and the expression level of *CXCL5*, respectively, in five of the cell lines and five osteosarcoma tumour samples. Figure 8 shows the DNA methylation and mRNA expression levels of *CXCL5*, and gel pictures of the methylation-specific PCR products are shown in Figure S7. For the cell lines, the PCR and microarray data correlated well. The microarray data indicated that IOR/OS14 was the only cell line not hyper-methylated compared to the osteoblasts, but the PCR data showed that *CXCL5* was partially methylated also in this cell line, as well as in the osteoblasts (Figure S7). Partial or full methylation of the investigated CpG island in the promoter region was identified in all cell lines and tumour samples, and all samples except OS94 showed under-expression.

**Figure 9:**
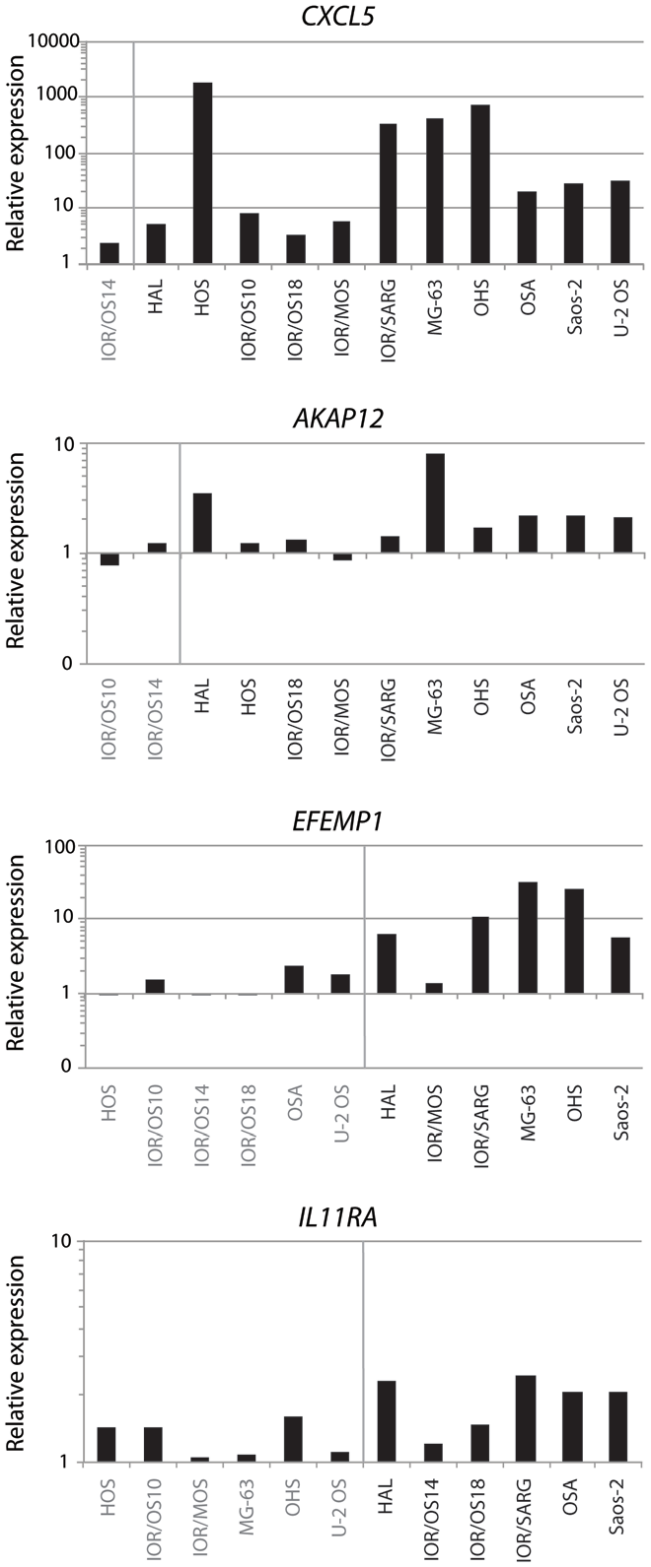
Gene expression after demethylation treatment. Relative gene expression levels of the frequently hyper-methylated and under-expressed genes *CXCL5*, *AKAP12*, *EFEMP1* and *IL11RA* after treatment with the demethylating agent 5-Aza-2′-deoxycytidine in 12 of the cell lines. The cell lines with hyper-methylation and under-expression of the genes are colour-coded in black, whereas gray colour indicates no aberrations in DNA methylation. The expression level without treatment has been set to 1 for each cell line.

To validate a causal association between hyper-methylation and under-expression, DNA methylation was removed by culturing the cells in a medium containing 5-Aza-2′-deoxycytidine and the effect on gene expression levels investigated. Four genes being frequently hyper-methylated and under-expressed were selected; *CXCL5* (18/19 cell lines), A kinase (PRKA) anchor protein 12 (*AKAP12*) (14/19 cell lines), EGF containing fibulin-like extracellular matrix protein 1 (*EFEMP1*) (10/19 cell lines) and interleukin 11 receptor, alpha (*IL11RA*) (10/19 cell lines). Twelve of the cell lines were treated with 5-Aza-2′-deoxycytidine, and the gene expression level of these four genes with and without treatment was determined by quantitative real-time RT-PCR, as shown in Figure 9.

The extent of reactivation of gene expression varied between the cell lines. *CXCL5*, which showed most frequent hyper-methylation and under-expression (18/19 cell lines), was reactivated in all of the tested cell lines, with two cell lines showing >100-fold increased expression level. The only cell line that was not hyper-methylated and under-expressed, IOR/OS14, showed the lowest level of increased expression (2–3 fold). For the genes *EFEMP1* and *AKAP12*, five and two cell lines showed >2-fold increased expression level, respectively, while only a low effect was observed for *IL11RA*. The genes that showed reactivation of expression were initially hyper-methylated in the affected cell lines, thus the demethylation treatment did not seem to affect the expression levels in general.

**Figure 10:**
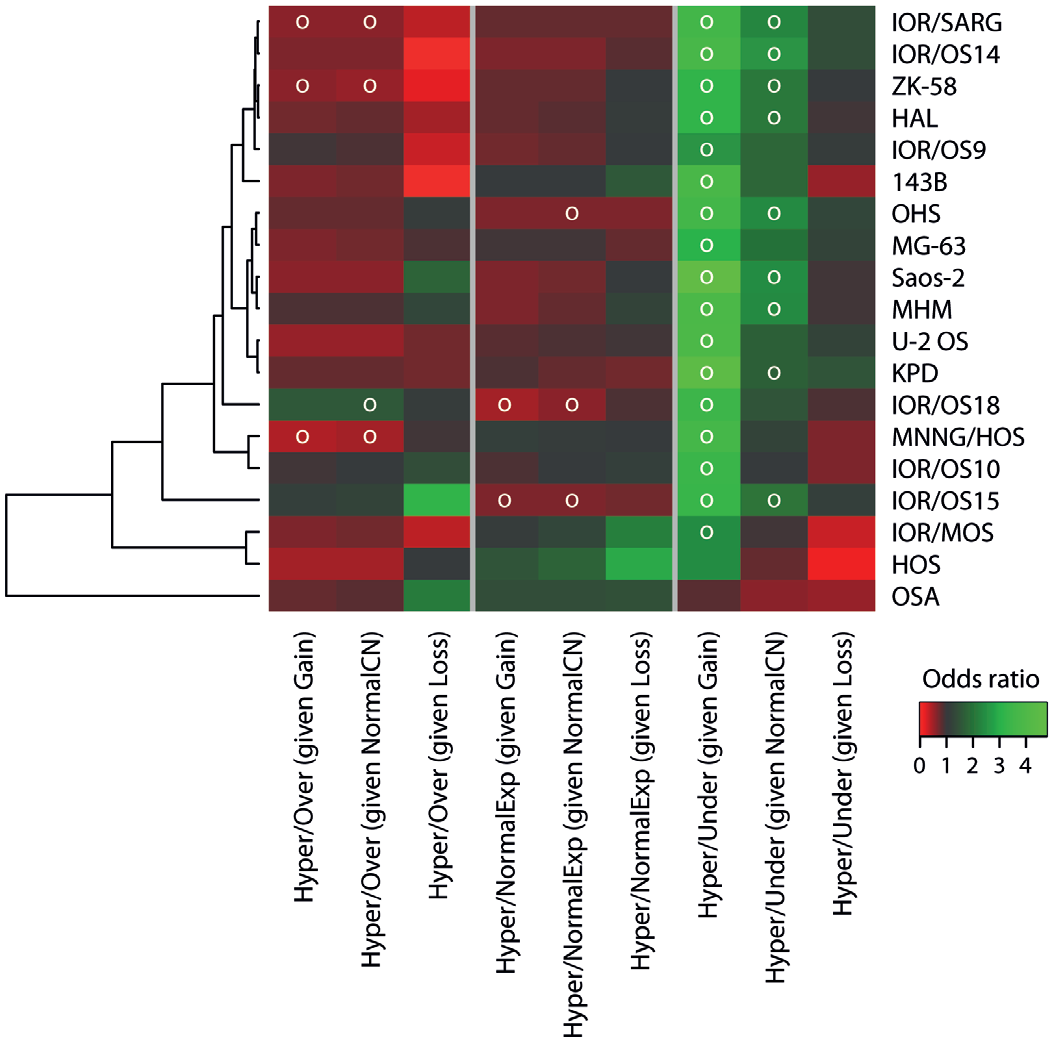
Data dependencies for three-way combinations. Heat map plots visualising the odds ratio and significance of data dependencies, with unsupervised hierarchical clustering of the cell lines, for combinations of hyper-methylation and mRNA expression, conditioning on the DNA copy number status. The colours of the heat map plot represent the odds ratio for a gene of having one type of aberration given that it has another type of aberration. Green, positive association (odds ratio >1); black, no association (odds ratio = 1) and red, negative association (odds ratio <1). A white circle indicates significance (Benjamini & Hochberg-corrected chi-square p-value <0.05). Exp, mRNA expression; CN, DNA copy number.

A heat map visualising the odds ratios and significance of data dependencies for different combinations of hyper-methylation and expression conditioning on the copy number state is shown in Figure 10. The significance was determined using Bonferroni-corrected chi-square p-values (p-value <0.05). The alterations were divided into three states for the DNA copy number (gain, normal and loss) and mRNA expression data (over-expression, normal and under-expression). Regardless of the DNA copy number state, no significant dependency was found, except for a few samples that showed dependencies between hyper-methylation and either normal or over-expression. However, there was a positive association of hyper-methylation and under-expression for genes with gain, and all cell lines, except two, showed significant dependencies of this combination. For some samples, there was also a positive association and significant dependency between hyper-methylation and under-expression for genes with normal copy number, but not for genes with loss.

Plots combining DNA copy number, DNA methylation and mRNA expression levels for the 16 genes that showed gain, hyper-methylation and under-expression in at least 6/19 cell lines are shown in Figure S8. The levels of methylation and expression anti-correlated in general quite well, but there were no clear differences in the pattern of methylation and expression levels between the cell lines with gain or normal copy number/loss for these genes.

## Discussion

Cell lines are valuable model systems when studying cancer biology, especially for rare tumours like osteosarcomas, where material from clinical samples is scarce. The EuroBoNeT panel of 19 osteosarcoma cell lines used here has previously been characterised by many means, including genetic, phenotypic and functional characterisation, and the cell lines reflect well many properties of osteosarcoma tumours [21], [22], [23]. Thus, the cell line panel constitutes a highly valuable model system for analyses of genetic and epigenetic aberrations in osteosarcomas.

In line with most conventional osteosarcomas, the cell lines showed a vast number of DNA copy number changes, reflecting the extreme genetic instability hallmarking high-grade osteosarcoma. Although recurrent alterations have been reported for almost every chromosome in osteosarcoma, gain of regions in 6p, 8q and 17p and loss of regions in 13q are most frequently reported [9], [10], [11], [12]. These regions were recurrently altered in more than 50% of the cell lines (Figure S2). High-level amplification was found in 6p and 8q, as well as in 1q, which has also been previously reported [11], [12], [25], [26]. When performing an unsupervised hierarchical clustering based on the DNA copy number profiles of the 19 cell lines and 32 osteosarcoma clinical samples [27], the cell lines were not systematically different from the clinical samples and all samples clustered intermingled (data not shown). All together, the results suggest that these cell lines are representative for osteosarcoma clinical samples in terms of DNA copy number changes.

Although the cell lines showed slightly more frequent regions of gain than loss, the number of genes with gain was far higher than the number of genes with loss for most cell lines (Figure 2A and Table S2). Genome-wide analysis using The Genomic HyperBrowser showed that regions with high frequencies of gain were significantly associated with gene-rich regions of the genome and conversely regions with high frequencies of loss with gene-poor regions. There was a significant association both at the genome-wide level and for most individual chromosome arms (Figure S3 and S4). A similar analysis of 3,131 cancer specimens belonging to several histological types demonstrated that deletions showed a bias towards regions of low gene density, whereas no association was observed for amplifications [28]. This indicates that the association of gain and gene-rich areas observed here is special for osteosarcoma, or perhaps detectable because of the unusually high number of amplified regions. Since gaining one copy gives less relative change of gene dosage than losing one, and also does not remove functional germ-line or somatic gene variation, it seems likely that initial loss of regions is on average more detrimental than gain. A general advantage of gains is that parts of an originally gained region that is disadvantageous may subsequently be lost, in this way generating the smaller, more focussed amplicons observed around some typical oncogenes, or the expression of passenger genes may be down-regulated by other mechanisms. For losses, on the other hand, regaining lost sequences is more complicated, as it requires additional rearrangement of the intact chromosome copy, and loss of heterozygosity would be maintained.

Since osteosarcomas have so many aberrations, a majority of these are most likely due to general instability of the genome. Recently, a new mechanism for genetic instability in cancer cells has been described, termed chromothripsis, in which a single chromosome is fragmented and then reassembled [29]. Chromothripsis has been suggested to occur in 2–3% of cancers, but the phenomenon has been observed in 25% of osteosarcoma and chordoma samples, affecting several chromosomes [29]. However, it seems likely that many of the genomic aberrations do not provide any advantage and may represent just genomic “noise”. Such noise would be expected to be better tolerated in gene-poor regions, but cannot explain the enrichment of gains in gene-rich regions, which appears to be oncogenically more relevant.

In contrast to DNA copy number profiles, mRNA expression profiles are more dynamic and may be more influenced by cell culturing and growth conditions. However, the comparison with normal osteoblast cultures rather than bone tissue should cancel most of the effects of *in vitro* growth. In previous work, it was shown that mRNA expression profiles characteristic of the histological subtypes of primary high-grade osteosarcoma clinical samples are preserved in these cell lines [23], indicating that they are representative of the primary tumour from which they are derived.

Although the understanding of epigenetic regulation of specific genes in osteosarcomas is increasing [30], [31], little is so far known about the global DNA methylation patterns. The cell lines showed in general more genes with hyper-methylation than hypo-methylation. Previous studies have shown that promoter-associated CpG islands are frequently hyper-methylated in cancer, whereas global hypo-methylation is often seen in gene-poor areas of the genome (reviewed in [32]). In line with the results here, previous investigations using Me-DIP-chips showed more hyper-methylation than hypo-methylation events in osteosarcoma tumours and cell lines compared to normal osteoblasts [19], [20]. Interestingly, there was in general an inverse relationship between the number of genes with hyper- and hypo-methylation, as opposed to the DNA copy number and mRNA expression (Figure 2). In contrast to the DNA copy number, there seemed to be a relationship between the number of genes with hyper- and hypo-methylation and the clustering pattern. However, no associations were found between the DNA copy number, DNA methylation and mRNA expression levels of the methyltransferases *DNMT1, -3A* and *-3B* (Figure S1) and the number of hyper- and hypo-methylated genes for the individual cell lines (Figure 2 and Table S2).

Functional enrichment analyses of the differentially methylated and expressed genes, respectively (Table 1, 2, S5 and S6), showed common terms like embryonic organ development and morphogenesis. However, the overlap between the lists was limited, supporting the notion that DNA methylation is only one among several mechanisms influencing gene expression.

Although the genetic and epigenetic profiling data provide valuable information on their own, an integrative approach may facilitate the identification of key genes and regulatory mechanisms involved in tumour development. A method originally used for the same type of data and for osteosarcoma cell lines [19] was adopted and further developed. In that study [19], cut-offs of intensity ratios were used to identify genes with aberrations in DNA copy number, DNA methylation and mRNA expression for individual samples, and Venn (two-way/three-way) analysis was used to select genes that showed alterations in more than one type of data. This approach was further improved by using the greater number of cell lines analysed here to filter the gene list obtained by Venn analysis based on recurrence. In this way, only genes that have the same two-way alteration in at least a certain number of the samples were identified. In addition, statistical tests for individual samples and heatmap visualisations were used to evaluate dependencies of types of data.

To examine how the different types of aberrations relate to each other, the odds ratios and significance of data dependencies for the 12 two-way combinations were identified for each cell line (Figure 7). There was a significant dependency between DNA copy number and mRNA expression, and to some extent between DNA methylation and mRNA expression, particularly for the combination hyper-methylation and under-expression. However, there was no dependency between the DNA copy number and DNA methylation, although the combination showing the highest number of recurrently altered genes was gain and hyper-methylation (Figure 3). Similar studies investigating five osteosarcoma tumours and two cell lines, respectively, showed strong correlation between gain and over-expression, loss and under-expression as well as gain and hypo-methylation [19], [20]. In contrast to the results presented here, few genes showed gain and hyper-methylation. A reason why no dependency between gain and hypo-methylation was observed in this study may be the low number of hypo-methylated genes detected.

Genes for which an altered expression level was consistent with aberrations in DNA copy number or DNA methylation (gain/over-expression, hypo-methylation/over-expression, loss/under-expression and hyper-methylation/under-expression) were identified for each cell line. Since different mechanisms for alteration of a certain pathway may be involved in each cell line, a test looking for genes significantly altered in all cell lines might not detect samples with defects in the same pathway, because the specific genes affected may vary. On the other hand, a recurrence threshold is needed to filter out noise or sample-specific events and to identify pathogenic alterations of general importance. A gene aberration frequency threshold of six or more cell lines (>30%) was chosen, giving a total of 335 recurrently altered genes. Interestingly, only 11 of these 335 genes showed simultaneous aberrations in both DNA copy number and DNA methylation. In addition, only 15 additional genes were identified when allowing genes with over-expression to be either gained or hypo-methylated and genes with under-expression to be either lost or hyper-methylated in a total of six or more cell lines, increasing the total number to 350 genes (Figure 6 and Table S7). This suggests that the expression levels of most genes with two types of aberrations, including aberrant mRNA expression, are regulated by alterations in either DNA copy number or DNA methylation, or conversely, that these mechanisms alter the activity of different subsets of genes. However, there will be other mechanisms like point mutations, loss of heterozygosity, nucleosome occupancy, microRNA (miRNA) or transcription factor regulation that also influence the mRNA expression levels.

By selecting genes with recurrent alterations in at least two of the three types of data, a list of genes involved in important biological functions and in a limited number of critical pathways was identified. Based on functional enrichment analysis, the most striking biological processes were development of the embryonic skeletal system and remodelling of the extracellular matrix (Table 3). A similar study of osteosarcoma showed that the most significant gene network, based on cumulative changes in DNA copy number, DNA methylation and mRNA expression, contained genes involved in organ and cellular development [20]. Although terms involving embryonic skeletal system were also identified using the list of differentially expressed genes from the comparison of the cell lines and the normal osteoblasts, the top cluster from that comparison contained general terms like translational elongation and ribosome (Table 2). The terms associated with the list of 350 genes that recurrently showed two types of aberrations seem highly relevant for osteosarcoma tumourigenesis, highlighting the significance of combining different types of data to identify important molecular markers and pathways involved.

Among the 159 recurrently gained and over-expressed genes, the most frequent were eukaryotic translation elongation factor 1 alpha 2 (*EEF1A2*, 13/19 cell lines), NADH dehydrogenase (ubiquinone) 1 beta subcomplex, 9, 22 kDa (*NDUFB9*, 13/19), ribophorin II (*RPN2*, 12/19) and cystathionine-beta-synthase (*CBS*, 12/19). Fifty-one of these 159 genes were also gained and over-expressed in >6/29 osteosarcoma clinical samples based on identical types of microarray data [27]. Among these was *NDUFB9*, gained and over-expressed in 13 of the 29 clinical samples. *NDUFB9* is located in 8q24.13 and is an accessory subunit of the mitochondrial membrane respiratory chain NADH dehydrogenase (Complex I), whereas *CBS* is a folate-metabolising gene located in 21q22.3. So far, little is known about a possible role for these genes in cancer development. *EEF1A2* and *RPN2* are both located in 20q, and *EEF1A2* has been suggested to be an oncogene and a diagnostic marker in various cancers [33], but has to our knowledge not previously been linked to sarcomas, whereas *RPN2* has been shown to confer drug resistance in breast cancer [34].

Another frequently gained and over-expressed gene was *RUNX2* (6/19 cell lines), which is a transcription factor essential for osteoblast maturation and bone development [35]. The aberrations of *RUNX2* were validated in five of the cell lines and five osteosarcoma tumour samples using quantitative real-time PCR and RT-PCR (Figure 5). None of the tumour samples showed gain of *RUNX2*, in contrast to the cell lines, but all showed increased expression at similar levels as the cell lines showing over-expression. *RUNX2* was also shown to be recurrently gained and over-expressed in 12/29 osteosarcoma clinical samples based on identical types of microarray data [27]. *RUNX2* is frequently amplified and over-expressed in osteosarcomas, and may play an important role in osteosarcoma tumourigenesis (reviewed in [35]).

Functional enrichment analysis of the 159 recurrently gained and over-expressed genes identified terms like embryonic skeletal system development and homeodomain in the first clusters (Table S9). HOX and other homeobox genes have crucial roles in development, and a number of these were gained and over-expressed, as has been frequently reported for other cancers (reviewed in [36]). Some of the gained HOX genes were also hyper-methylated, and did not show increased expression, whereas several others were hyper-methylated, but only one recurrently under-expressed (*MSX1*) (Table S3).

The homeobox gene *DLX5* was recurrently gained and over-expressed (7/19 cell lines), and this transcription factor interacts with bone morphogenetic protein (BMP) signalling and is involved in bone and cartilage development (reviewed in [37]). The aberrations of *DLX5* were validated in five of the cell lines and five osteosarcoma tumour samples using quantitative real-time PCR and RT-PCR (Figure 5). As for *RUNX2*, none of the tumour samples showed gain of *DLX5*, but all showed increased expression at similar levels as the cell lines showing over-expression. *DLX5* was also shown to be recurrently gained and over-expressed in 7/29 osteosarcoma clinical samples based on identical types of microarray data [27], and *DLX5* was part of a gene expression prediction profile that could distinguish different histological subtypes of osteosarcoma, being down-regulated in fibroblastic osteosarcoma [23]. *DLX5* has also been shown to be differentially methylated and under-expressed in enchondromas from patients with Ollier disease, which is a non-hereditary skeletal disorder [38].

Among the nine recurrently hypo-methylated and over-expressed genes was the gene “preferentially expressed antigen in melanoma” (*PRAME*, 11/19 cell lines), which is over-expressed and a prognostic marker for clinical outcome in various types of cancers [39]. Four of the cell lines showed simultaneously gain and two additional cell lines showed gain and over-expression. *PRAME* was also over-expressed in 12/29 osteosarcoma clinical samples based on identical types of microarray data (no methylation data was available) [27], and has also recently been shown by others to be over-expressed in osteosarcomas [40], [41]. Hypo-methylation of *PRAME* has been demonstrated to be responsible for the increased expression in various types of cancer [42].

Among the 158 recurrently hyper-methylated and under-expressed genes, the most frequent were “mesoderm specific transcript homolog (mouse)” (*MEST*), neuronatin (*NNAT*) and *CXCL5*, all altered in 18/19 cell lines. *MEST* and *CXCL5* were also shown to be under-expressed in 29/29 osteosarcoma clinical samples, respectively, whereas *NNAT* was under-expressed in 27/29 samples, based on identical types of microarray data (no methylation data was available) [27]. Both *MEST* and *NNAT* are imprinted genes, and *MEST* has been shown to be down-regulated in a model of human osteosarcoma, suggesting a role in tumourigenesis [43]. Consistent with the results here, loss of expression of *NNAT* has been associated with promoter hypermethylation in pituitary adenoma [44].

The frequent hyper-methylation and under-expression of *CXCL5* were validated in five of the cell lines and five osteosarcoma tumour samples using methylation-specific PCR and quantitative real-time RT-PCR, respectively (Figure 8 and S7). Partial or full methylation of the investigated CpG island in the promoter region was identified in all cell lines, and partial methylation was also identified in all tumour samples and the normal osteoblasts. Although methylation-specific PCR is not a quantitatively accurate method, the amount of PCR products indicated a higher degree of methylation of *CXCL5* in all cell lines and two of the tumour samples compared to the normal osteoblasts (Figure S7). Furthermore, all samples except one tumour sample showed under-expression. For *CXCL5*, previous reports are more equivocal, showing up-regulation correlated to poor survival in colorectal and pancreatic cancer [45], [46], whereas another study showed correlation with under-expression of *CXCL5* and poor survival for colorectal cancer [47]. Demethylation using 5-Aza-2′-deoxycytidine showed that *CXCL5* was reactivated in all cell lines tested, with two cell lines showing more than 100-fold increased expression level (Figure 9). Tumour-specific methylation of *CXCL5* has also been observed in 80% of primary lung adenocarcinomas and 65% of lung adenocarcinoma cell lines [48], and demethylation using 5-Aza-2′-deoxycytidine also restored the expression of *CXCL5* [48]. Similar results were observed here for the other genes tested, supporting that the low expression levels of these genes are indeed caused by promoter hyper-methylation.

The significantly differentially methylated genes between the cell lines and the normal osteoblasts, which were all hyper-methylated, were enriched for terms like skeletal system development and homeodomain (Table 1), similar to the genes showing gain and over-expression (Table S9). Based on this, it seems like the methylation pattern reflects turning off a tissue-specific epigenetic program. However, the 158 genes that were both hyper-methylated and under-expressed were enriched for more general terms like signal peptide and extracellular matrix in the first clusters from the functional enrichment analysis (Table S10).

Genes with gain showed a positive association between hyper-methylation and under-expression (Figure 10), and the most common three-way combination was gain, hyper-methylation and under-expression (16 recurrently altered genes in >6/19 cell lines). This suggests that hyper-methylation of passenger genes in gained regions may be advantageous, conceivably because it counteracts the effect of over-expression of detrimental genes. Two of these 16 genes, S100 calcium binding protein A16 (*S100A16*) and maternally expressed 3 (non-protein coding) (*MEG3*), were also gained and under-expressed in 7 and 6 of 29 osteosarcoma clinical samples, respectively, based on identical types of microarray data (no methylation data was available) [27]. An integrative genomic analysis of familial breast tumours has also revealed frequent hyper-methylation of genes that showed copy number gain [49], and genes with copy number gains, low expression and high methylation levels have been identified in urothelial carcinomas by integrative analysis [50]. However, no consistent differences in the pattern of methylation and expression for the 16 recurrently altered genes were found when cell lines with gain were compared with those with normal copy number or loss (Figure S8). This suggests that methylation is not directly related to the amplification or deletion processes. In another study of osteosarcoma, gained and hyper-methylated genes showed far more over- than under-expression [20]. However, although a comparable number of genes showed recurrent gain, hyper-methylation and over-expression (12 recurrently altered genes in >6/19 cell lines), there was no significant dependency of this combination in these cell lines.

In summary, integrative analysis of genome-wide genetic and epigenetic alterations identified dependencies and relationships between DNA copy number, DNA methylation and mRNA expression in osteosarcomas. For the samples investigated, novel correlations between DNA copy number alterations and gene density were identified. The recurrently altered genes with two types of aberrations, including aberrant mRNA levels, showed in general alterations in either DNA copy number or DNA methylation, both within individual samples and across the sample panel. On the other hand, a positive association of gain with hyper-methylation and under-expression was observed, suggesting that hyper-methylation may oppose the effects of increased copy number for detrimental genes. This is especially an issue in osteosarcomas, which is highly genetically unstable, thereby suffering from many disadvantageous genomic aberrations that may be compensated for by other mechanisms. The analyses revealed a number of genes regulated by alterations in DNA copy number and DNA methylation, and additional experiments are needed to investigate their potential role in osteosarcoma development. The results show the importance of combining different types of molecular data to better comprehend the biology of osteosarcoma.

## Materials and Methods

### Osteosarcoma Cell Lines

Nineteen osteosarcoma cell lines collected within EuroBoNeT (http://www.eurobonet.eu) [21] were analysed. Four cell lines were established at the Norwegian Radium Hospital (HAL, KPD, MHM and OHS) and seven were established at the Istituto Ortopedico Rizzoli (IOR/OS9, IOR/OS10, IOR/OS14, IOR/OS15, IOR/OS18, IOR/MOS and IOR/SARG). The cell line ZK-58 [51] was kindly provided by Dr. Karl-Ludwig Schäfer, Düsseldorf, Germany. The cell lines 143B, HOS, MNNG/HOS, MG-63, OSA (SJSA-1), Saos-2 and U-2 OS were obtained from ATCC (http://www.lgcstandards-atcc.org). The cell lines 143B and MNNG/HOS are derived from the HOS cell line. Cell line authentication was performed by DNA profiling using short tandem repeats (STR) using Powerplex 16 (Promega, Madison, USA), and the data was validated using the profiles of the EuroBoNeT cell line bank [21] and ATCC. Data for all cell lines are given in Table S1.

The cells were grown in RPMI1640 (Lonza, Basel, Switzerland) or DMEM (Lonza) supplemented with 10% foetal calf serum (PAA Laboratories GmbH, Pasching, Austria), GlutaMAX (Life Technologies, California, USA) and penicillin/streptomycin (Lonza), at 37°C with 5% CO_2_. All cells were split when reaching 80% confluency.

### Osteosarcoma Tumour Samples

Five human sarcomas classified as conventional osteosarcomas were selected from a tumour collection at the Department of Tumor Biology at the Norwegian Radium Hospital. All tumors were diagnosed according to the current World Health Organization classification [1]. Tumour samples were collected immediately after surgery, cut into small pieces, frozen in liquid nitrogen and stored at −70°C until use. The clinical information was retrieved from the MEDinsight database at the Norwegian Radium Hospital. Data for all tumour samples are given in Table S1.

### Normal Samples

Four normal bone samples and two osteoblast cultures were used as normal controls. Two normal bone samples were obtained from cancer patients (one with osteosarcoma and one with renal cell carcinoma) at the Norwegian Radium Hospital. The normal bone was collected as distant as possible from the tumour site, and SNP arrays confirmed that these samples had normal DNA copy number. Two additional normal bone samples from different donors were purchased from Capital Biosciences (Maryland, USA). Two primary osteoblast cultures isolated from human calvaria of different donors were purchased from ScienCell Research Laboratories (California, USA). Data for all normal samples are given in Table S1.

The osteoblast cells were maintained in medium provided by the manufacturer, split when reaching 80% confluency, and harvested when enough cells for DNA and RNA isolation were obtained.

### Ethics Statement

The information given to the patients, the written consent used, the collection of samples and the research project were approved by the ethical committee of Southern Norway (Project S-06133).

### Array CGH

DNA was isolated using the Wizard Genomic DNA Purification Kit (Promega). High-resolution array CGH was performed using the Affymetrix Genome-Wide Human SNP Array 6.0 (Affymetrix, California, USA), containing more than 1.8 million SNPs, according to the manufacturer’s protocol. Quality control was performed using the Genotyping Console v3.0.1 software (Affymetrix), applying the contrast quality control (CQC) algorithm with a minimal call rate of >86%. DNA copy number analysis was performed using the Nexus software (BioDiscovery, California, USA), with the SNPRank segmentation algorithm using default settings (threshold of 0.6 for high copy gain, 0.2 for gain, −0.2 for loss and −1.0 for homozygous loss). The categories high copy gain and gain were combined, as well as the categories loss and homozygous loss. For each cell line, tab separated text files with probe intensities, as well as copy number states for each gene, were exported for further analysis. The frequency plot of DNA copy number changes was made using Nexus. The SNP array dataset has been deposited in the Gene Expression Omnibus (GEO) data repository (www.ncbi.nlm.nih.gov/geo/, accession number GSE36003, SuperSeries number GSE36004).

### DNA Methylation Profiling

DNA methylation profiling of approximately 27,000 CpG sites across the genome was performed using the Illumina HumanMethylation27 BeadChip (Illumina Inc., California, USA) according to the manufacturer’s protocol. The array is used to estimate the level of methylation of the CpG sites. Data extraction and initial quality control of the bead summary raw data were performed using BeadStudio v3.1.0.0 and the Methylation module v1.9, both provided by Illumina. For each cell line and normal sample, tab separated text files with avgBeta (average ratio of signal from methylated probe relative to the sum of both methylated and unmethylated probes) values for each probe was exported for further analysis. The DNA methylation dataset has been deposited in the GEO data repository (www.ncbi.nlm.nih.gov/geo/, accession number GSE36002, SuperSeries number GSE36004).

### mRNA Expression Profiling

RNA was isolated using the standard TRIzol procedure (Life Technologies), and further purified with an RNeasy mini column (QIAGEN GmbH, Hilden, Germany), according to the manufacturers’ instructions. The purity and quantity of the extracted RNA were measured using the NanoDrop ND1000 spectrophotometer (Nanodrop Technologies, Delaware, USA), and the RNA integrity was evaluated using the Agilent 2100 Bioanalyzer and the RNA nano 6000 kit (Agilent Technologies Inc., California, USA).

mRNA expression profiling was performed using the Illumina HumanWG-6 v2 Expression BeadChip according to the manufacturer’s protocol as previously described [52]. Data extraction and initial quality control of the bead summary raw data were performed using BeadStudio v3.1.0.0 from Illumina and the Gene Expression module v3.1.7. Variance-stabilizing transformation (vst) [53] and quantile normalisation were performed using the R package lumi, which is part of the Bioconductor project (http://www.R-project.org) [54]. The vst is almost identical to a log_2_ transformation, only differing at the lower end of intensities where the vst transformed values are slightly higher than the log_2_ transformed values. The data were annotated using the HumanWG-6_V2_R4_11223189_A annotation file from Illumina. For each cell line and normal sample, tab separated text files with vst transformed and quantile normalised intensities for each probe were exported for further analysis. The mRNA expression dataset has been deposited in the GEO data repository (www.ncbi.nlm.nih.gov/geo/, accession number GSE36001, SuperSeries number GSE36004).

### Hierarchical Clustering

Unsupervised hierarchical clustering of all three data types was performed in R v.2.13.0, using the method complete linkage and Spearman correlation as distance measure. For the DNA copy number, the DNA methylation and the mRNA expression data, the probe intensities, avgBeta probe values and vst transformed and quantile normalised probe intensities, respectively, were used to calculate distances.

### Identification of Alterations within Each Sample

The copy number, methylation and expression data were exported as tab separated text files from their respective native software. DNA copy number changes were identified using Nexus as previously described, assigning each gene with a copy number event (gain, normal or loss). Alterations in DNA methylation were identified by calculating the ratio between avgBeta probe values of the individual cell lines and the average of the controls (normal osteoblasts), deltaBeta. The thresholds used to define probes showing hyper-methylation and hypo-methylation were deltaBeta >0.4 and < −0.4, respectively. Alterations in mRNA expression were identified by calculating the ratio between vst transformed and quantile normalised probe intensities of the individual cell lines and the average of the controls (normal osteoblasts). The thresholds used to define probes showing over-expression and under-expression were vst ratio >1 and < −1, respectively. The probes were collapsed to gene level for the analyses, keeping the probe level information.

Six tab separated text files in total with binary scores (0 for no alteration and 1 for alteration) for the copy number (gain and loss), methylation (hyper-methylation and hypo-methylation) and expression (over-expression and under-expression) data for all genes were generated for each cell line using R scripts (available upon request). In cases where the probes for a gene showed different values and subsequently were assigned to different categories, the gene name was included in all the corresponding lists.

### Comparison of Copy Number Frequency and Gene Distribution

A “.bgr” (bedgraph) file was exported from Nexus for use in genome browsers. DNA copy number gain frequency data were imported as a “marked.bed” data type into the The Genomic Hyperbrowser (http://hyperbrowser.uio.no/hb/) [24], and analysed against UCSC known genes. Using these two tracks as input, the test “Higher values in segments” was used, testing whether gain frequency data (number of copy number gains for a given region) are higher in regions of genes than expected by chance. p-values were computed by Monte Carlo, using 1,000 MC samples. The underlying null hypothesis was that the gain value of a region, and the overlap with genes falling within the region, are uncorrelated. The test statistics used was the mean gain value inside regions covered by genes, and Monte Carlo estimates were computed by randomly permuting gain values (keeping the same segments as in the original gain track, but shuffling the gain values associated to these segments). The same analysis was performed on copy number loss frequency, except that the alternative hypothesis was that copy number values were lower instead of higher than expected.

### Identification of Differentially Methylated and Expressed Genes

The Bioconductor packages Lumi, Limma and MethyLumi were used to perform t-tests between the osteosarcoma cell lines and normal osteoblasts to identify significantly differentially methylated and expressed genes, respectively. In MethyLumi, M-values (log_2_ ratio of methylated probe intensity and unmethylated probe intensity) were calculated and used to perform the t-tests. Separate lists with differentially methylated and expressed genes, with a Benjamini & Hochberg-corrected p-value <0.05 and absolute value of fold change >6 for the methylation data and >0.5 for the expression data, were used for functional enrichment analysis.

### Functional Enrichment Analysis

The functional annotation tool of DAVID (Database for Annotation, Visualization and Integrated Discovery, developed by NIAID/NIH, http://david.abcc.ncifcrf.gov/home.jsp) [55], [56] was used for functional enrichment analysis, with the DAVID default population background for *Homo sapiens* (for the gene lists from the integration analyses, the 11,843 genes from chromosome 1–22 common to the three microarray platforms were used as background). Genes were uploaded as Illumina probe IDs to avoid using official gene symbols that may be mapped ambiguously, as recommended by DAVID. For the methylation data, genes had to be uploaded using official gene symbols since DAVID does not permit Illumina methylation probe IDs for mapping. Default settings were used for the analyses.

### Integration of All Data Types and Identification of Recurrently Altered Genes

The 11,843 genes from chromosome 1–22 common to the three microarray platforms were used for the integration of the data. The six text files with binary scores were combined in order to identify genes with alterations in two types of data and to create contingency tables for each cell line using R scripts (available upon request). With two types of changes for each of the three data sets (gain and loss, hyper- and hypo-methylation, over- and under-expression), 12 two-way combinations were possible, whereas 8 three-way combinations were possible. A recurrence threshold of 6/19 cell lines (>30%) was used to identify recurrently altered genes with two types of aberrations, considering the combinations gain/over-expression, hypo-methylation/over-expression, loss/under-expression and hyper-methylation/under-expression.

### Data Type Dependencies

The contingency tables were used to evaluate data dependencies within each sample by calculating the odds ratio for the different two-way combinations of data, as well as the three-way combinations conditioning on the copy number state. The Bonferroni-corrected chi-square p-values of the combinations were also determined.

### Quantitative Real-time PCR and RT-PCR

Quantitative real-time PCR was performed using the 7900HT Fast Real-Time PCR System (Life Technologies). The copy numbers of the genes distal-less homeobox 5 (*DLX5*) and runt-related transcription factor 2 (*RUNX2*) were determined using TaqMan Copy Number Assays (assay ID Hs01209848_cn and Hs00753612_cn, respectively). The genes eukaryotic translation elongation factor 1 gamma (*EEF1G*) and F-box protein 11 (*FBXO11*) (assay ID Hs03771595_cn and Hs02528370_cn, respectively) were used as endogenous controls for normalisation. These two genes are located in 11q12.3 and 2p16.3, respectively, and showed low level of DNA copy number changes in a large panel of osteosarcoma samples ([27] and Kresse et al, unpublished). The copy number levels were determined using the CopyCaller Software v2.0 program (Life Technologies) as described by the manufacturer, and the average copy number of *EEF1G* and *FBXO11* was used for normalisation.

The High Capacity RNA-to-cDNA Master Mix (Life Technologies) was used for cDNA synthesis, and quantitative real-time reverse-transcription PCR (qRT-PCR) was performed using the 7900HT Fast Real-Time PCR System (Life Technologies). The expression levels of the genes distal-less homeobox 5 (*DLX5*), runt-related transcription factor 2 (*RUNX2*), chemokine (C-X-C motif) ligand 5 (*CXCL5*), A kinase (PRKA) anchor protein 12 (*AKAP12*), EGF containing fibulin-like extracellular matrix protein 1 (*EFEMP1*) and interleukin 11 receptor, alpha (*IL11RA*) were determined using TaqMan Gene Expression Assays (assay ID Hs00193291_m1, Hs01047976_m1, Hs00171085_m1, Hs00374507_m1, Hs00244575_m1 and Hs00234415_m1, respectively). The gene glyceraldehyde 3-phosphate dehydrogenase (*GAPDH*, assay ID Hs99999905_m1) was used as an endogenous control for normalisation. The relative expression levels were determined using the comparative C_T_ method as described by the manufacturer.

### Methylation-specific PCR

Genomic DNA was sodium bisulphite-treated as previously described [57]. Twenty-two ng of converted DNA was used to assess the methylation status of the *CXCL5* promoter by methylation-specific PCR. The primers were designed using Methyl Primer Express v1.0 (Life Technologies). Each bisulphite-treated DNA was amplified in a 50 µl reaction volume using the following primer sets; CXCL5_M_F (5′-TTAGGAATTCGCGATCGTTC-3′) and CXCL5_M_R (5′-CACCGCTAACGATAAACCCT-3′), as well as CXCL5_U_F (5′-AGTTTAGGAATTTGTGATTGTTT-3′) and CXCL5_U_R (5′-CACCACTAACAATAAACCCTAAC-3′). The forward primer covers four CpGs and overlaps with one of the two probes for *CXCL5* on the Illumina HumanMethylation27 BeadChip (probe ID cg10088985). The primers cover a total of six CpGs located in the first exon of *CXCL5*.

PCR was carried out using the EpiTect MSP kit (QIAGEN) with the following PCR conditions; 95°C for 10 min, followed by 94°C for 15 s, 53°C for 15 s and 72°C for 30s, for 40 cycles and a final extension step at 72°C for 10 min. The PCR products were separated by electrophoresis using a 2% agarose gel. Each sample was scored for the presence of PCR product for the methylated (M_F/M_R) and unmethylated (U_F/U_R) primer sets. The EpiTect PCR Control DNA Set (QIAGEN) was used for optimization and to confirm the specificity of the primers. DNase- and RNase-free water was used as a negative control.

### Demethylation Treatment

Twelve of the osteosarcoma cell lines were seeded at a density of 5,000–7,500 cells/cm^2^. The following day, the medium was replaced with a medium containing 1 µM of 5-Aza-2`-deoxycytidine (Sigma Aldrich, Montana, USA), which again was replenished every 24 hours. After three days, total RNA was isolated using the QIAGEN miRNeasy Mini Kit (QIAGEN) according to the manufacturer's protocol, prior to treatment with amplification-grade DNase I (Life Technologies) to avoid amplification of contaminating genomic DNA.

## Supporting Information

Figure S1 **Plots of DNA copy number, DNA methylation and mRNA expression levels for DNA methyltransferase genes.**
(PDF)

Figure S2 **Genome-wide frequency plot of DNA copy number.**
(PDF)

Figure S3 **Frequency plot of copy number aberrations and gene density for chromosome arms 2q, 8p, 19p and 19q.**
(PDF)

Figure S4 **Frequency plot of copy number aberrations and gene density for chromosome arms not significant for gain.**
(PDF)

Figure S5 **Plots of the number of common genes for individual aberration types and three-way combinations at different sample recurrence thresholds.**
(PDF)

Figure S6 **Hierarchical clustering of osteosarcoma cell lines based on expression level of 350 genes that recurrently showed two types of aberrations.**
(PDF)

Figure S7 **Gel pictures of PCR products from methylation-specific PCR.**
(PDF)

Figure S8 **Plots of DNA copy number, DNA methylation and mRNA expression levels for 16 recurrent genes with gain, hyper-methylation and under-expression.**
(PDF)

Table S1 **Clinical data for osteosarcoma cell lines, osteosarcoma tumour samples and normal samples.**
(PDF)

Table S2 **Number of genes with each type of aberration (DNA copy number, DNA methylation and mRNA expression).**
(PDF)

Table S3 **List of 328 significantly differentially methylated genes.**
(XLSX)

Table S4 **Top 10 clusters from enrichment analysis of differentially methylated genes in DAVID.**
(XLSX)

Table S5 **List of 283 significantly differentially expressed genes.**
(XLSX)

Table S6 **Top 10 clusters from enrichment analysis of differentially expressed genes in DAVID.**
(XLSX)

Table S7 **List of 350 genes that recurrently showed two types of aberrations.**
(XLSX)

Table S8 **Top 10 clusters from enrichment analysis of 350 genes that recurrently showed two types of aberrations in DAVID.**
(XLSX)

Table S9 **Top 10 clusters from enrichment analysis of 159 genes with gain and over-expression in DAVID.**
(XLSX)

Table S10 **Top 10 clusters from enrichment analysis of 158 genes with hyper-methylation and under-expression in DAVID.**
(XLSX)
