# Supplementary material for: Integrative Analysis Reveals Relationships of Genetic and Epigenetic Alterations in Osteosarcoma
Source: PLoS One. 2012 Nov 7;7(11):e48262. doi: 10.1371/journal.pone.0048262 (PMC3492335; doi:10.1371/journal.pone.0048262)

Figure S1. DNA copy number, DNA methylation and mRNA expression levels of DNA methyltransferase genes *DNMT1*, *-3A* and *-3B* (Kresse et al)

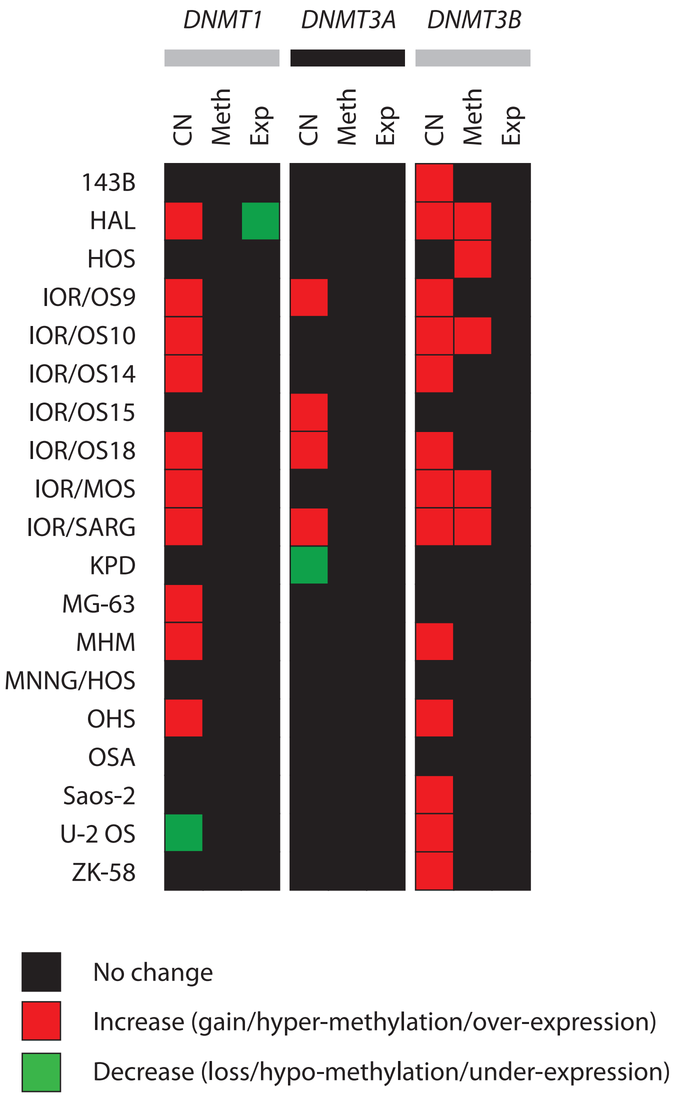

Supplement: Figure S1 — Plots of DNA copy number, DNA methylation and mRNA expression levels for DNA methyltransferase genes. (PDF) [file pone.0048262.s001.pdf]
