# Supplementary figures and images for: Integrative Analysis Reveals Relationships of Genetic and Epigenetic Alterations in Osteosarcoma
Source: PLoS One. 2012 Nov 7;7(11):e48262. doi: 10.1371/journal.pone.0048262 (PMC3492335; doi:10.1371/journal.pone.0048262)

**Figure S2.** Genome-wide frequency plot of DNA copy number (Kresse et al)

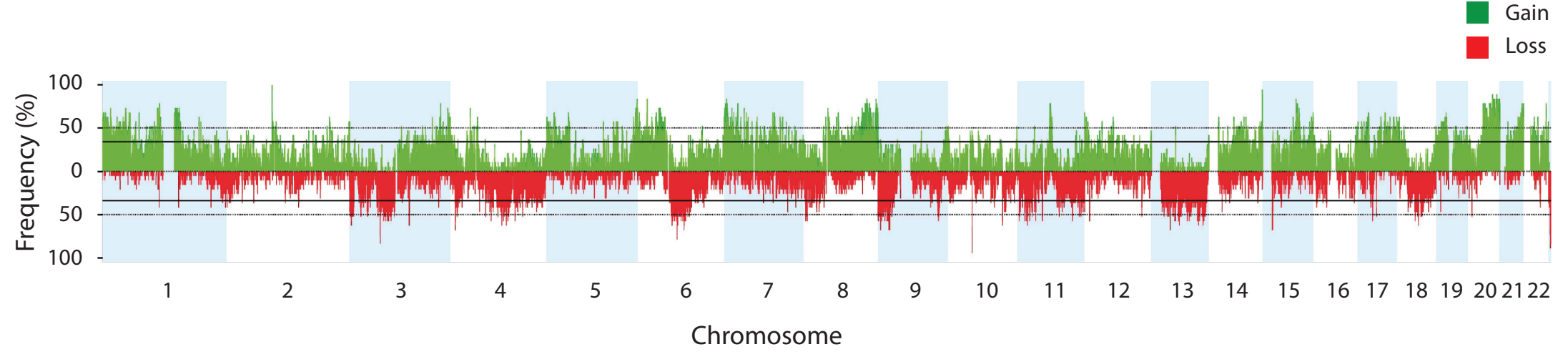

Supplement: Figure S2 — Genome-wide frequency plot of DNA copy number. (PDF) [file pone.0048262.s002.pdf]
