# Supplementary material for: Integrative Analysis Reveals Relationships of Genetic and Epigenetic Alterations in Osteosarcoma
Source: PLoS One. 2012 Nov 7;7(11):e48262. doi: 10.1371/journal.pone.0048262 (PMC3492335; doi:10.1371/journal.pone.0048262)

Figure S3. Frequency plot of DNA copy number aberrations and gene density for chromosome arms 2q, 8p, 19p and 19q (Kresse et al)

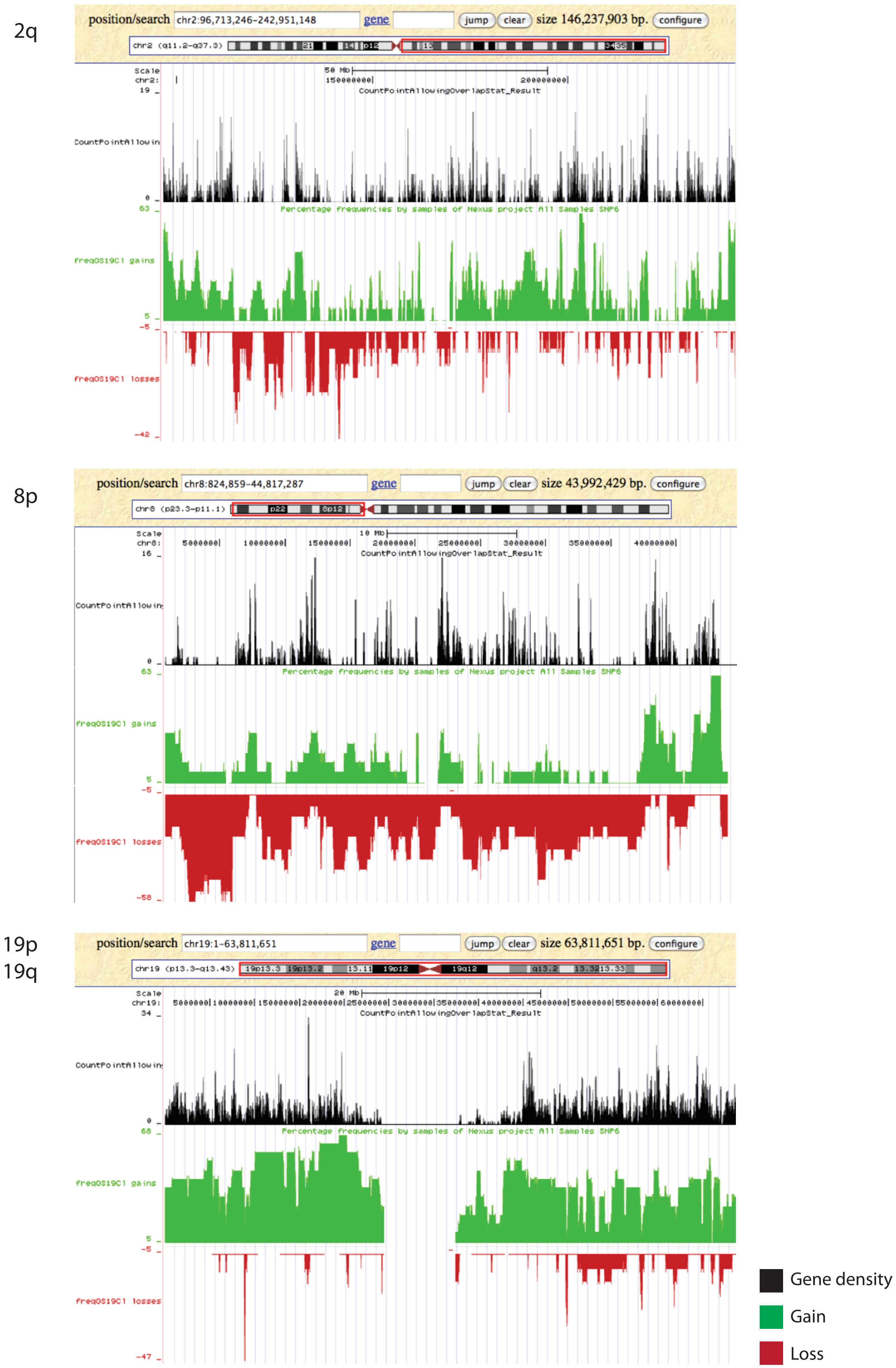

Supplement: Figure S3 — Frequency plot of copy number aberrations and gene density for chromosome arms 2q, 8p, 19p and 19q. (PDF) [file pone.0048262.s003.pdf]
