# Supplementary material for: Integrative Analysis Reveals Relationships of Genetic and Epigenetic Alterations in Osteosarcoma
Source: PLoS One. 2012 Nov 7;7(11):e48262. doi: 10.1371/journal.pone.0048262 (PMC3492335; doi:10.1371/journal.pone.0048262)
Supplement: Figure S4 — Frequency plot of copy number aberrations and gene density for chromosome arms not significant for gain. (PDF) [file pone.0048262.s004.pdf]

Figure S4. Frequency plot of DNA copy number aberrations and gene density for chromosome arms not significant for gain (Kresse et al)

3p

4p

4q

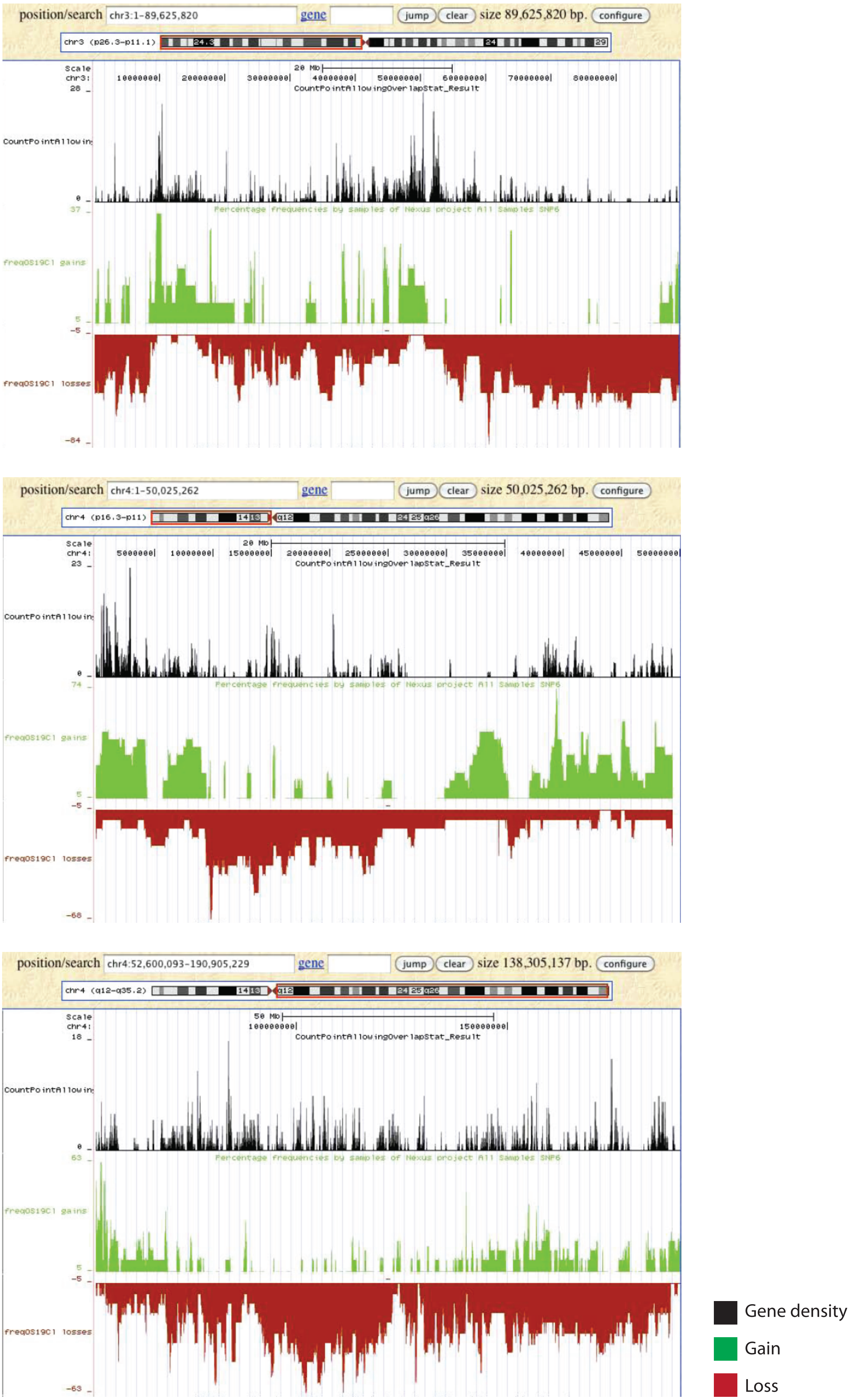

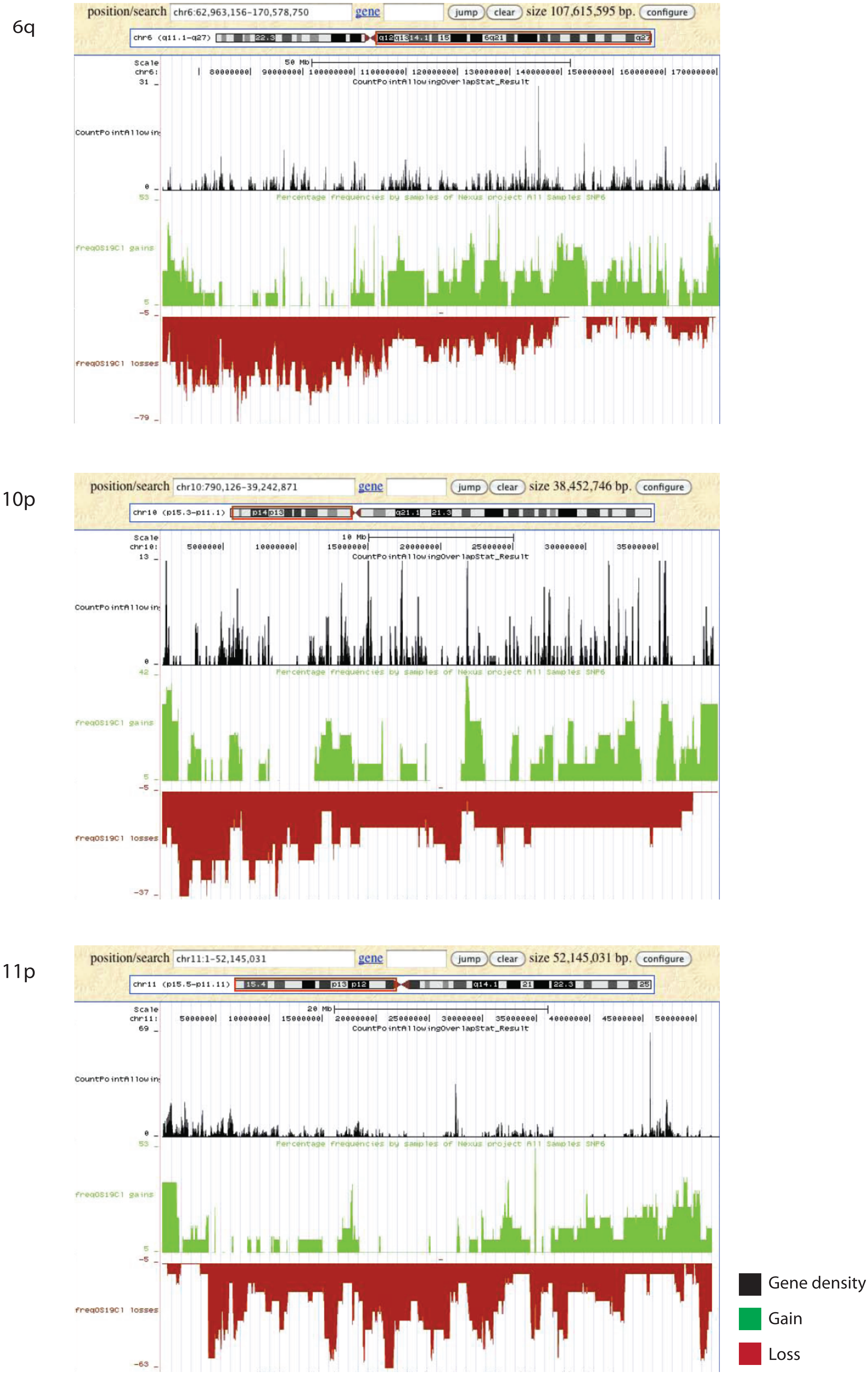
6q
position/search chr6:62,963,156-170,578,750
gene
jump
clear
size 107,615,595 bp.
configure
CountPointAllowingOverlapStat_Result
Percentage frequencies by samples of Nexus project All Samples SNP6
freqOS19C1 gains
freqOS19C1 losses
10p
position/search chr10:790,126-39,242,871
size 38,452,746 bp.
11p
position/search chr11:1-52,145,031
size 52,145,031 bp.
Gene density
Gain
Loss

12p

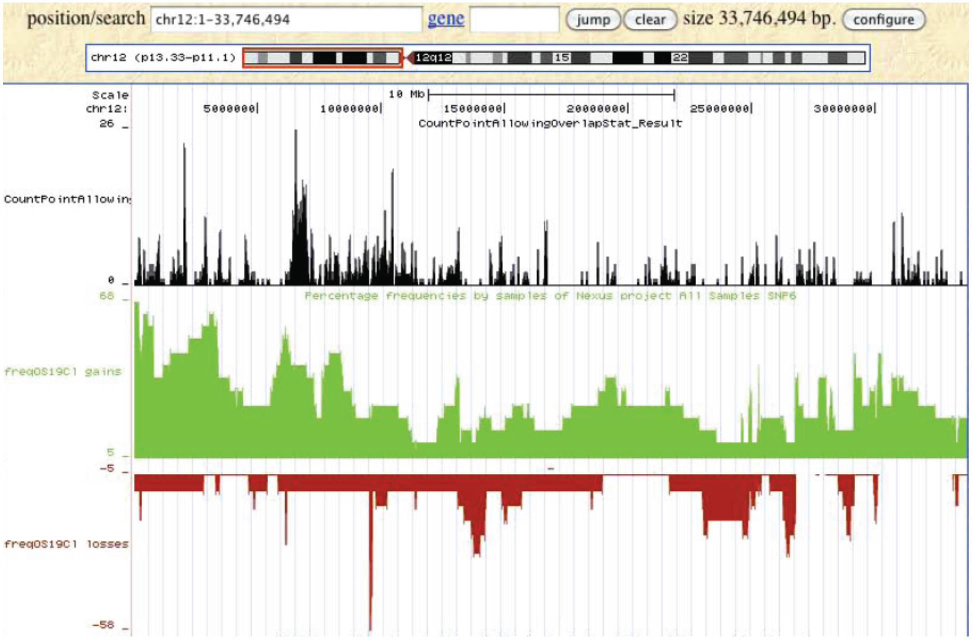

13q

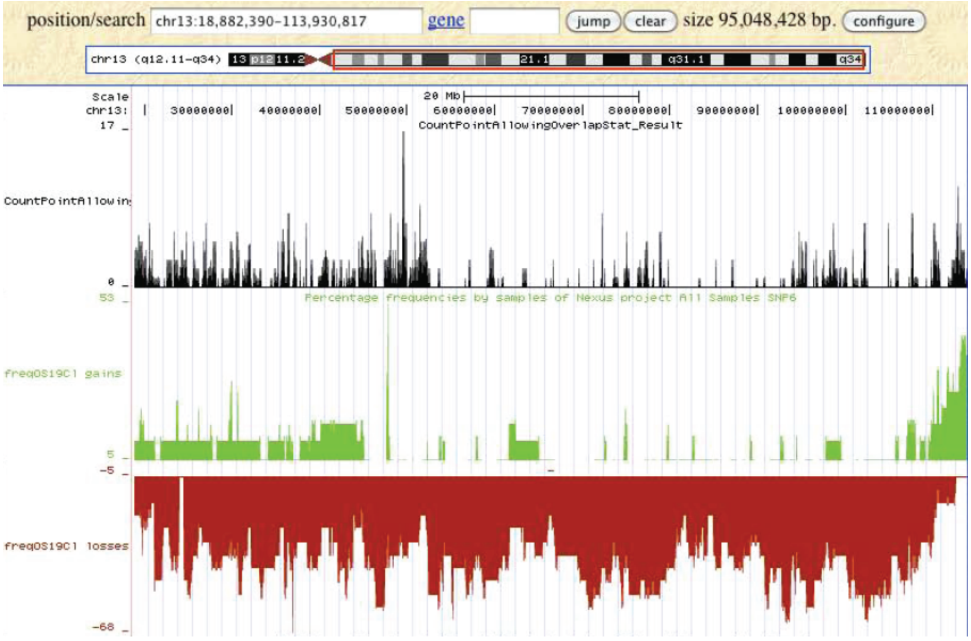

14q

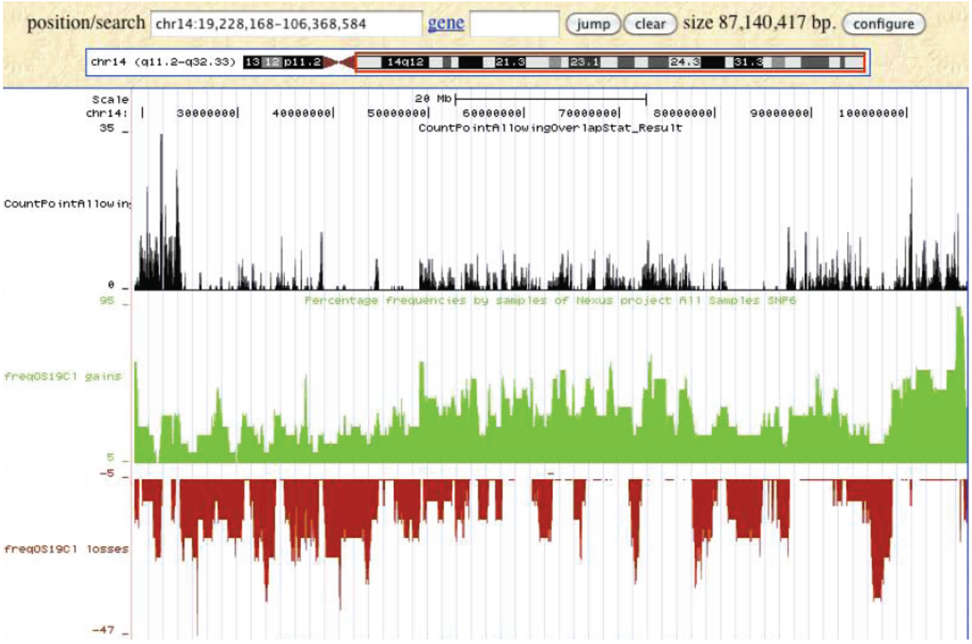

Gene density
Gain
Loss

17q

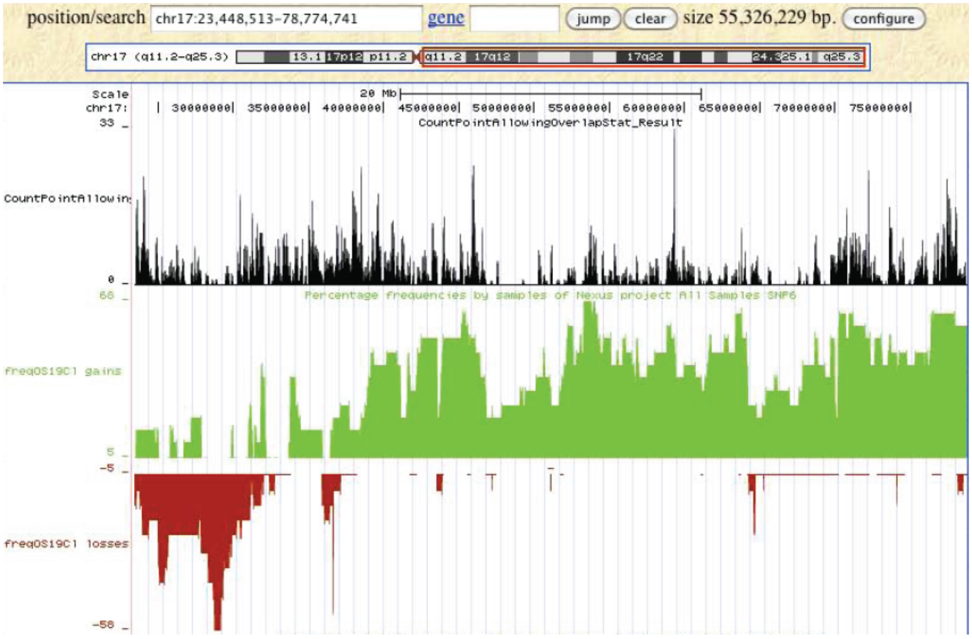

18p

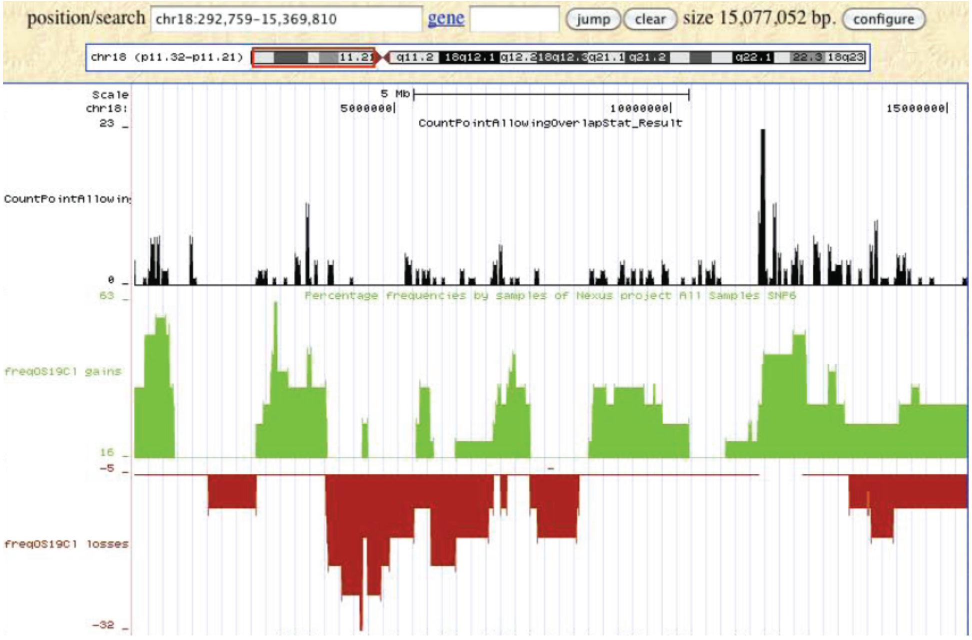

18q

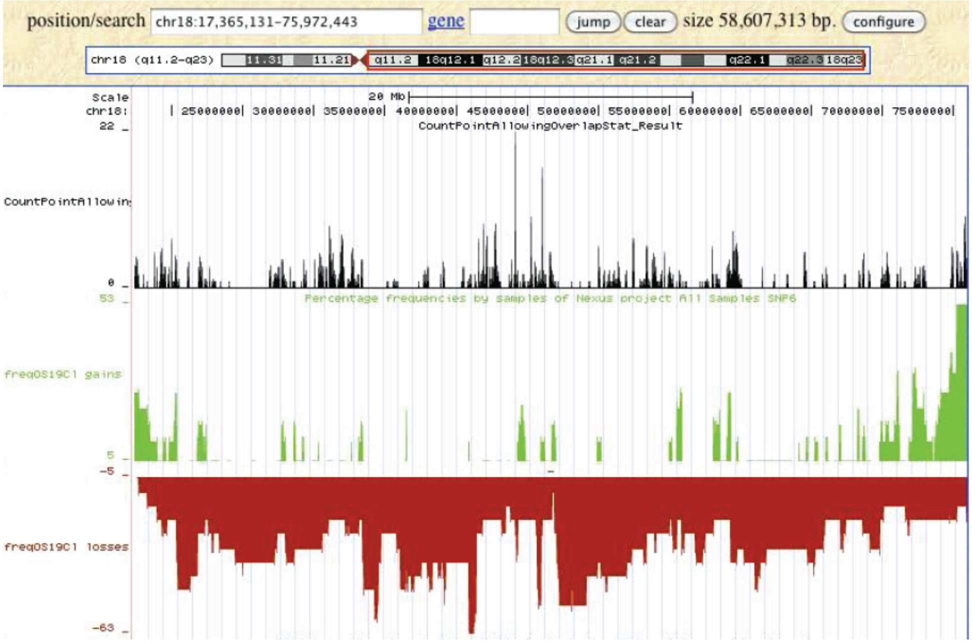

Gene density
Gain
Loss
