# Supplementary material for: Integrative Analysis Reveals Relationships of Genetic and Epigenetic Alterations in Osteosarcoma
Source: PLoS One. 2012 Nov 7;7(11):e48262. doi: 10.1371/journal.pone.0048262 (PMC3492335; doi:10.1371/journal.pone.0048262)

**Figure S5.** Number of common genes for all three-way combinations and the individual aberration types (Kresse et al)

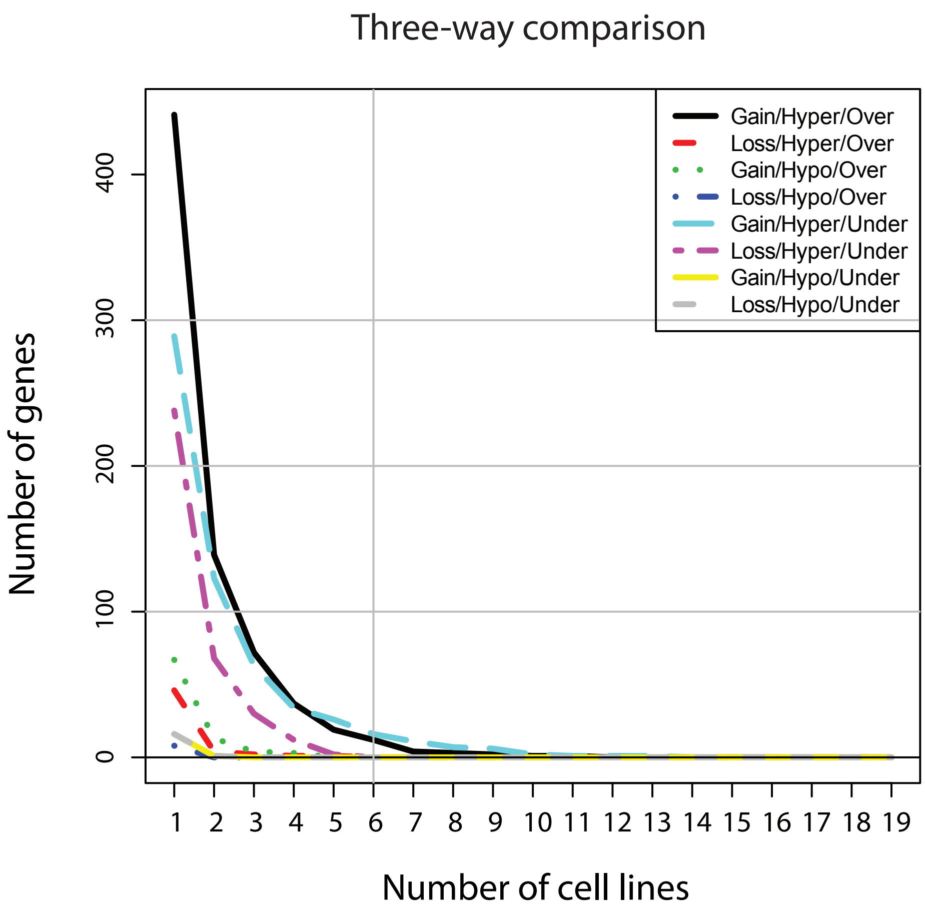


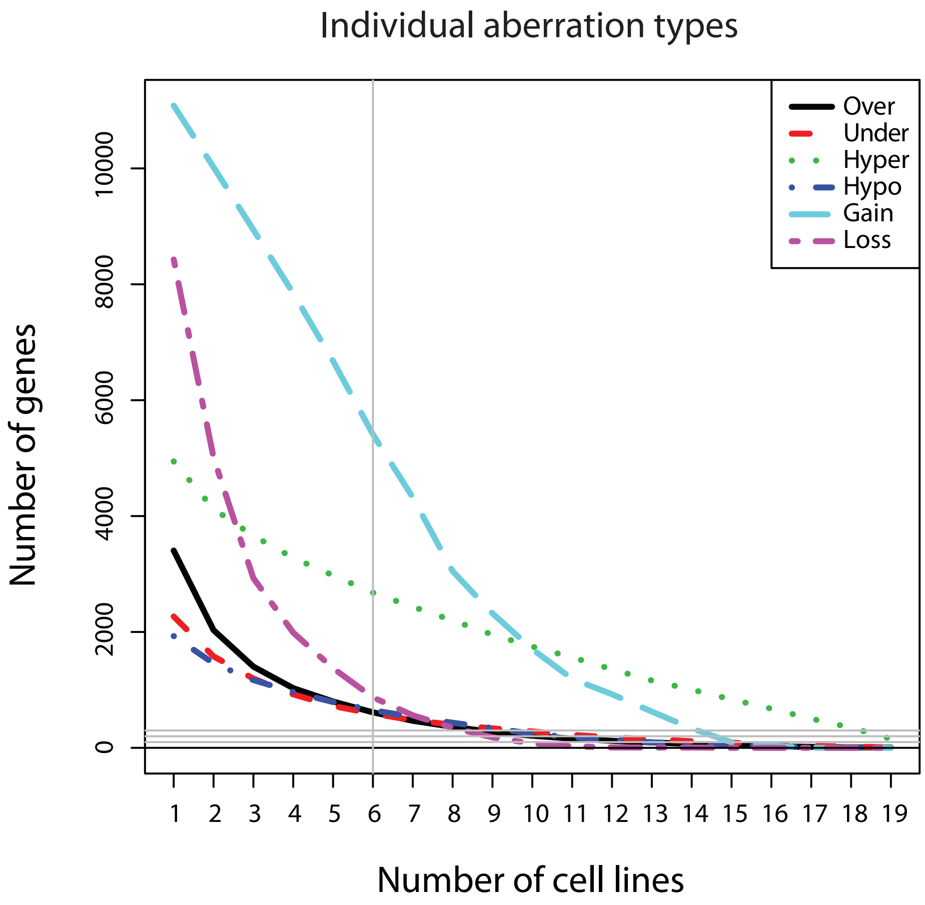

Supplement: Figure S5 — Plots of the number of common genes for individual aberration types and three-way combinations at different sample recurrence thresholds. (PDF) [file pone.0048262.s005.pdf]
