# Supplementary material for: Integrative Analysis Reveals Relationships of Genetic and Epigenetic Alterations in Osteosarcoma
Source: PLoS One. 2012 Nov 7;7(11):e48262. doi: 10.1371/journal.pone.0048262 (PMC3492335; doi:10.1371/journal.pone.0048262)

Figure S6. Hierarchical clustering of osteosarcoma cell lines based on expression level of 350 genes (some with multiple probes) that recurrently showed two types of aberrations (Kresse et al)

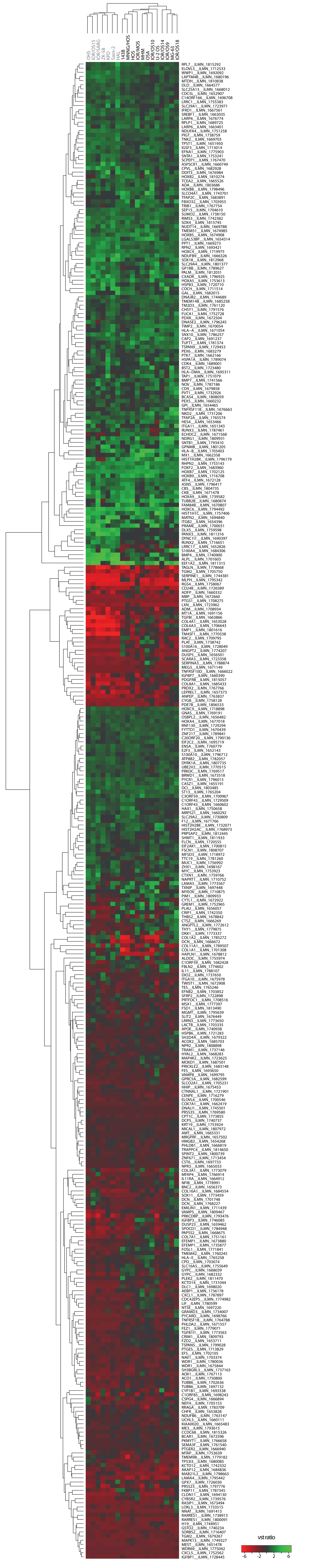

Supplement: Figure S6 — Hierarchical clustering of osteosarcoma cell lines based on expression level of 350 genes that recurrently showed two types of aberrations. (PDF) [file pone.0048262.s006.pdf]
