# Supplementary material for: Integrative Analysis Reveals Relationships of Genetic and Epigenetic Alterations in Osteosarcoma
Source: PLoS One. 2012 Nov 7;7(11):e48262. doi: 10.1371/journal.pone.0048262 (PMC3492335; doi:10.1371/journal.pone.0048262)
Supplement: Figure S7 — Gel pictures of PCR products from methylation-specific PCR. (PDF) [file pone.0048262.s007.pdf]

Figure S7. Gel pictures (normal and 3D) of PCR products from methylation-specific PCR of *CXCL5* in five cell lines, five tumour samples and two normal osteoblast samples. The PCR products show the expected size of 152 bp. M, methylated primer; U, unmethylated primer; Neg, negative control (Kresse et al)

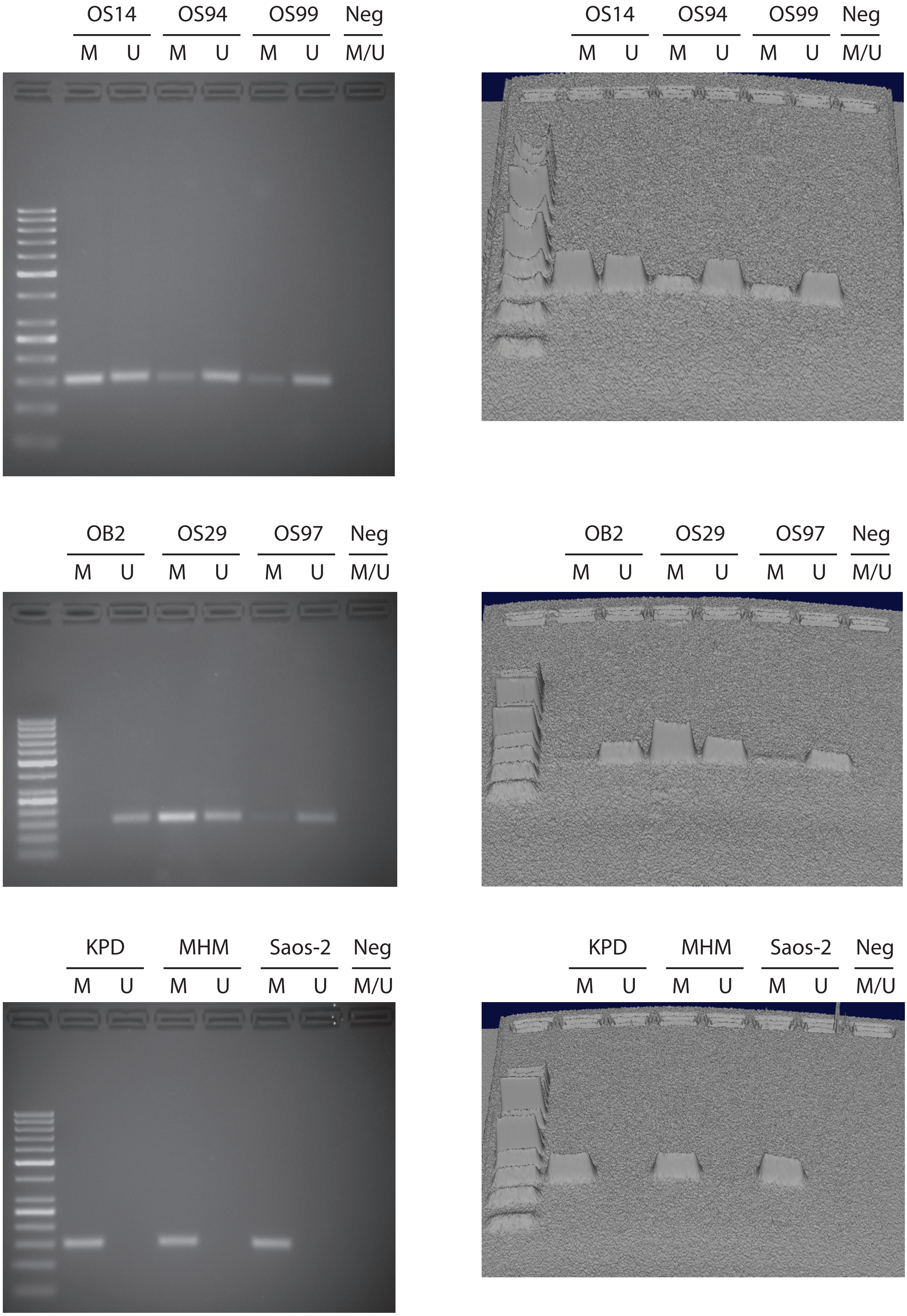

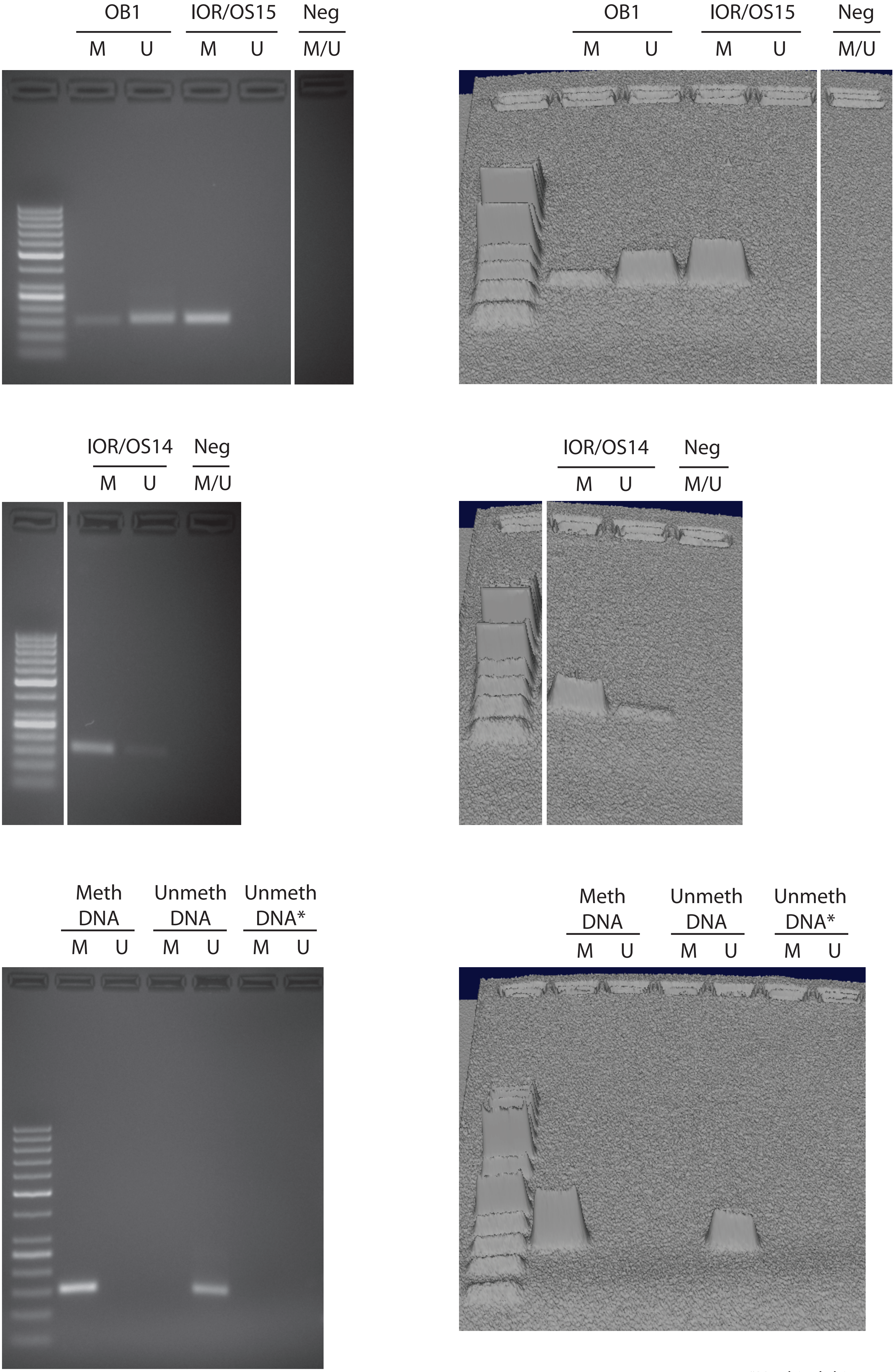

*Not bisulphite-treated
