# Supplementary material for: Integrative Analysis Reveals Relationships of Genetic and Epigenetic Alterations in Osteosarcoma
Source: PLoS One. 2012 Nov 7;7(11):e48262. doi: 10.1371/journal.pone.0048262 (PMC3492335; doi:10.1371/journal.pone.0048262)
Supplement: Figure S8 — Plots of DNA copy number, DNA methylation and mRNA expression levels for 16 recurrent genes with gain, hyper-methylation and under-expression. (PDF) [file pone.0048262.s008.pdf]

Figure S8. Plot of DNA copy number, DNA methylation and mRNA expression levels for 16 recurrent genes with gain, hyper-methylation and under-expression (Kresse et al)

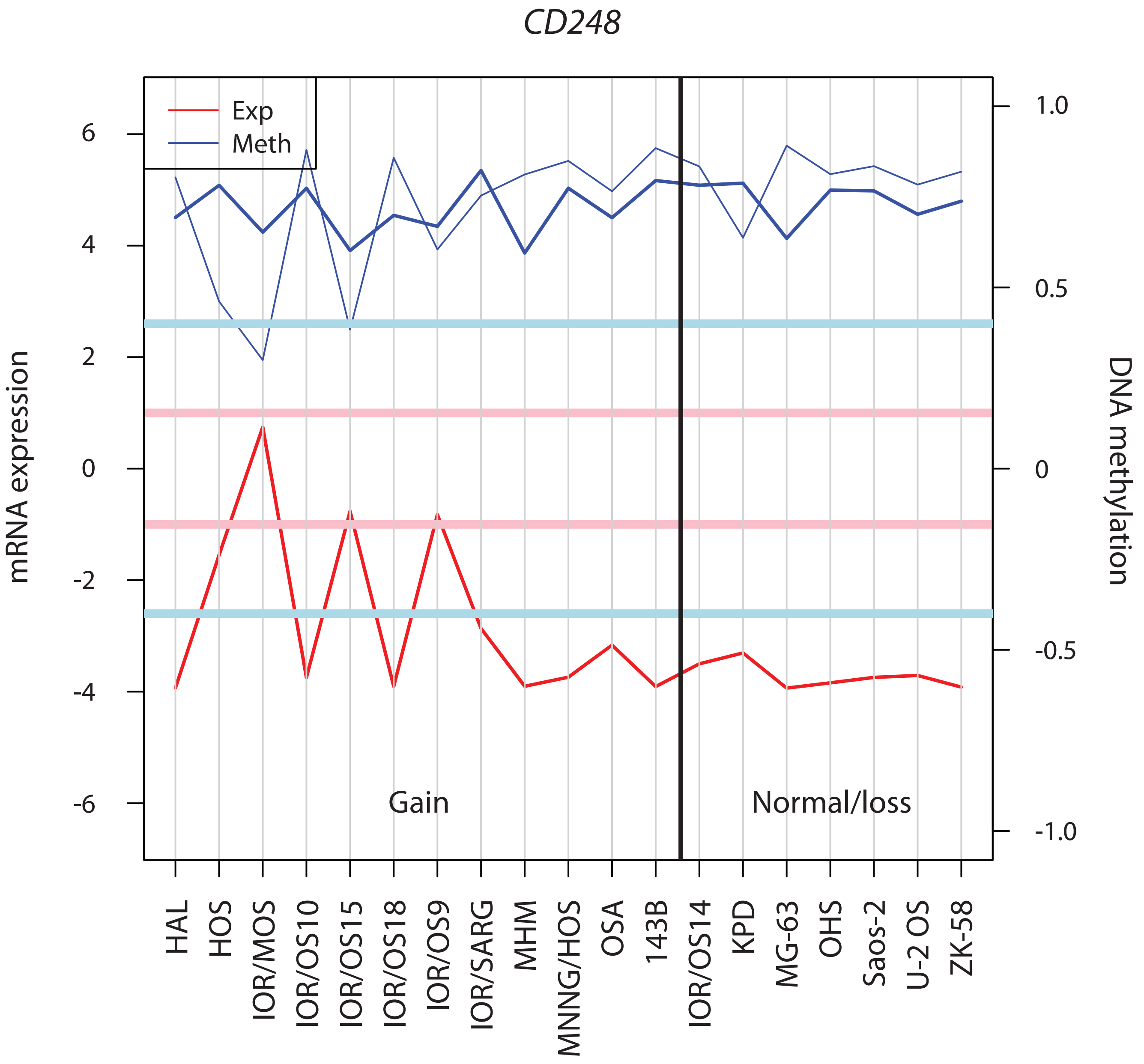

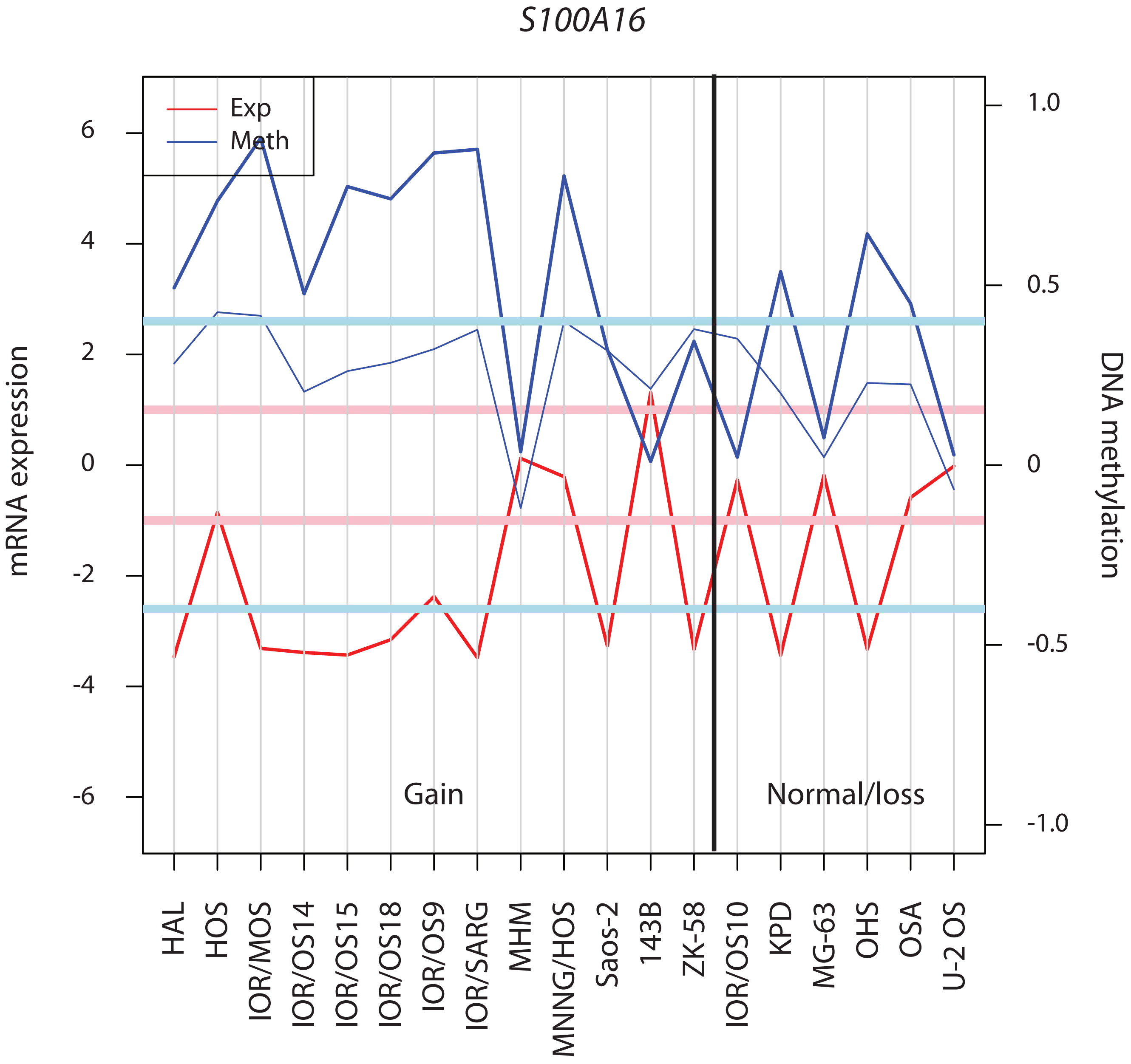

S100A16
Exp
Meth
mRNA expression
DNA methylation
6
4
2
0
-2
-4
-6
1.0
0.5
0
-0.5
-1.0
Gain
Normal/loss
HAL
HOS
IOR/MOS
IOR/OS14
IOR/OS15
IOR/OS18
IOR/OS9
IOR/SARG
MHM
MNNG/HOS
Saos-2
143B
ZK-58
IOR/OS10
KPD
MG-63
OHS
OSA
U-2 OS

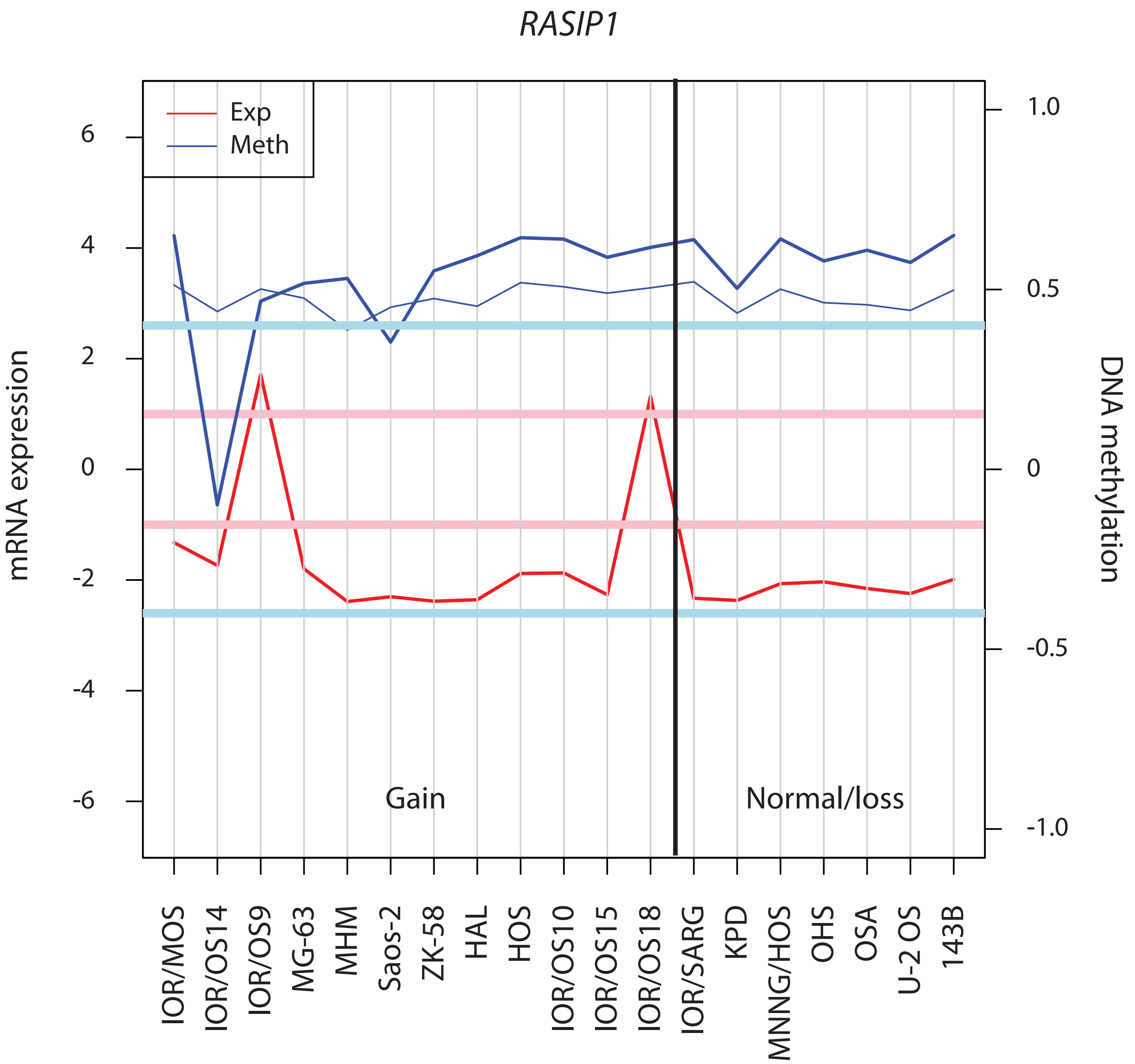

RASIP1
Exp
Meth
mRNA expression
DNA methylation
6
4
2
0
-2
-4
-6
1.0
0.5
0
-0.5
-1.0
Gain
Normal/loss
IOR/MOS
IOR/OS14
IOR/OS9
MG-63
MHM
Saos-2
ZK-58
HAL
HOS
IOR/OS10
IOR/OS15
IOR/OS18
IOR/SARG
KPD
MNNG/HOS
OHS
OSA
U-2 OS
143B

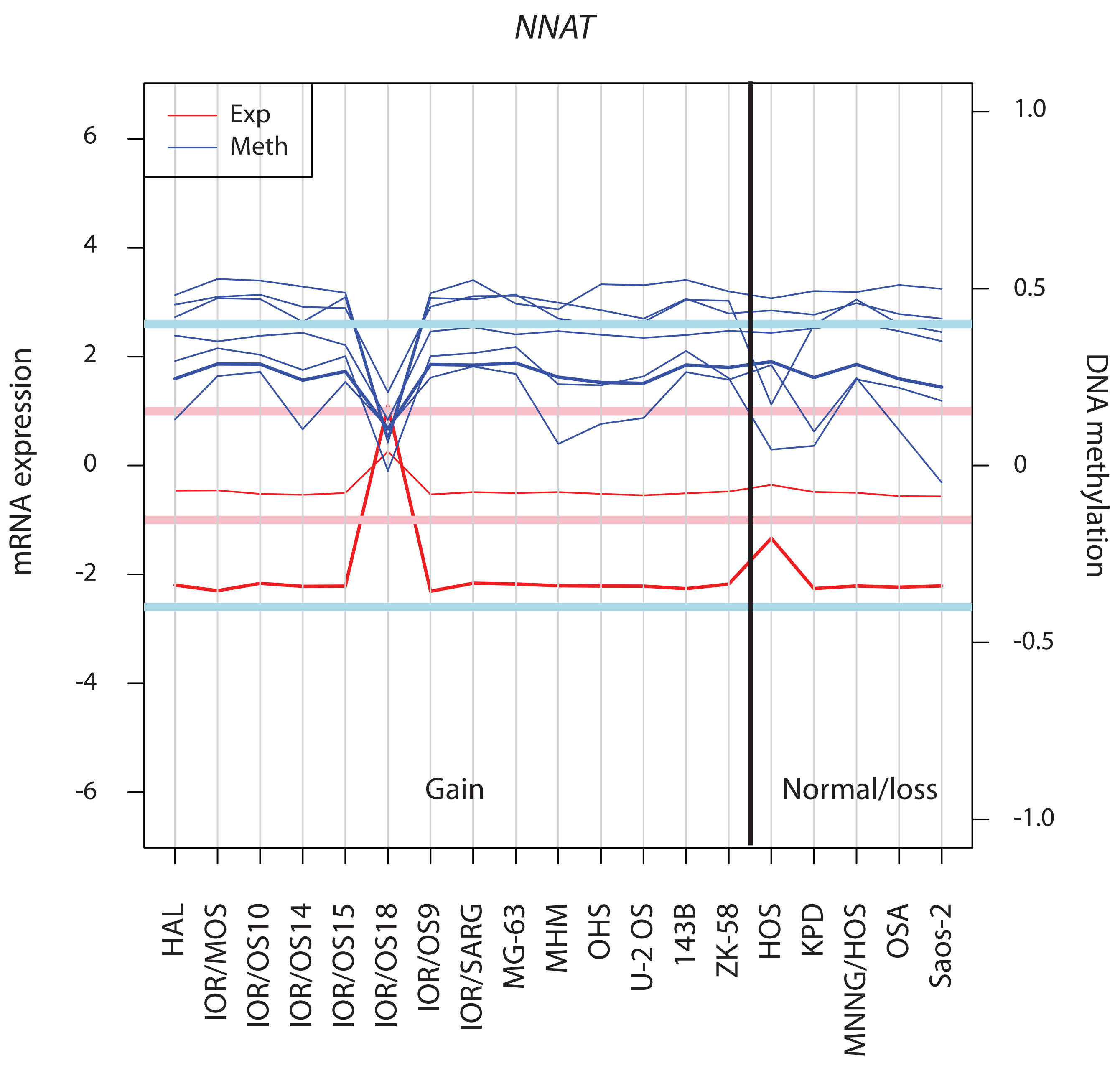

NNAT
Exp
Meth
mRNA expression
DNA methylation
6
4
2
0
-2
-4
-6
1.0
0.5
0
-0.5
-1.0
Gain
Normal/loss
HAL
IOR/MOS
IOR/OS10
IOR/OS14
IOR/OS15
IOR/OS18
IOR/OS9
IOR/SARG
MG-63
MHM
OHS
U-2 OS
143B
ZK-58
HOS
KPD
MNNG/HOS
OSA
Saos-2

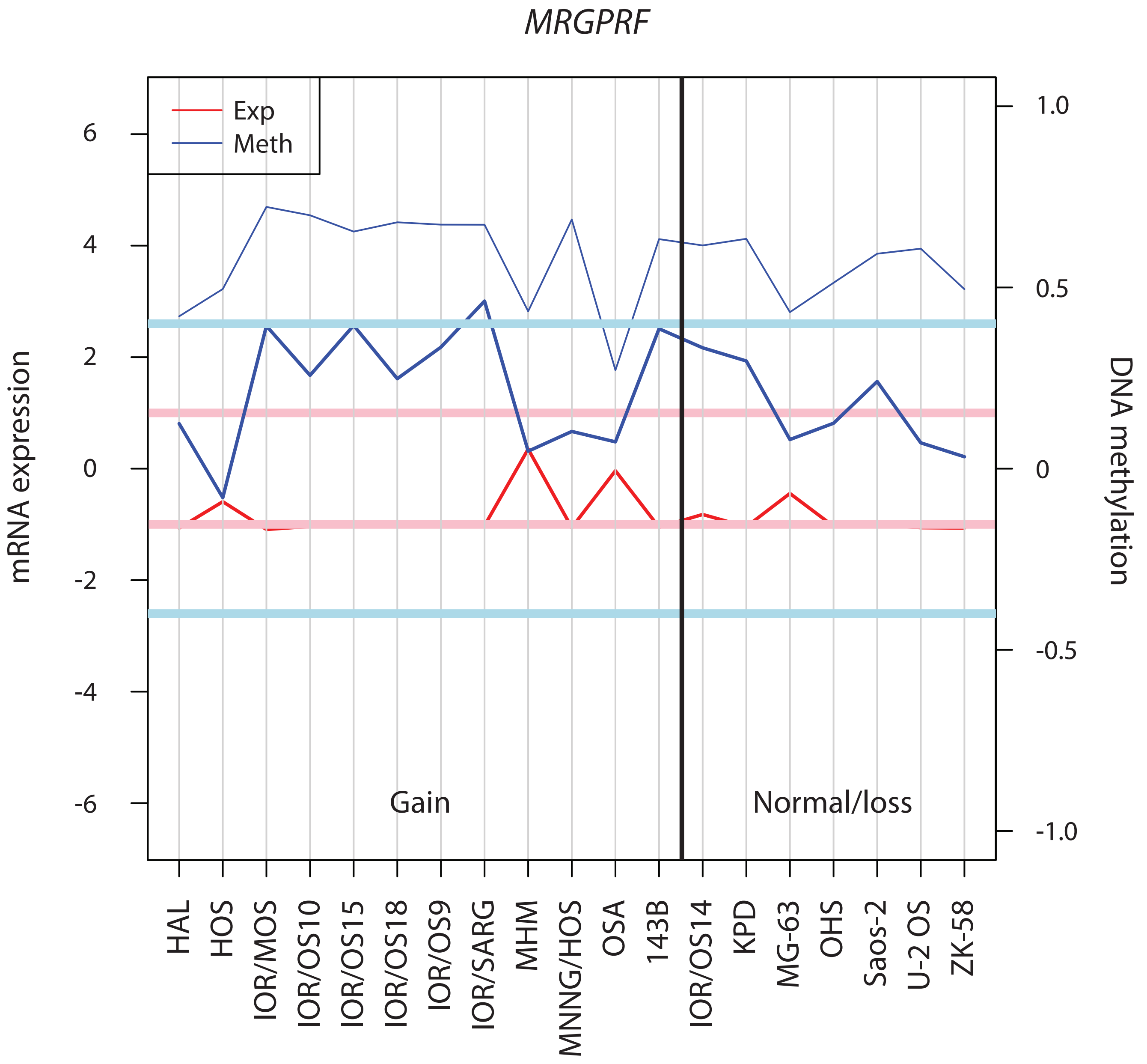

MRGPRF
Exp
Meth
mRNA expression
DNA methylation
6
4
2
0
-2
-4
-6
1.0
0.5
0
-0.5
-1.0
Gain
Normal/loss
HAL
HOS
IOR/MOS
IOR/OS10
IOR/OS15
IOR/OS18
IOR/OS9
IOR/SARG
MHM
MNNG/HOS
OSA
143B
IOR/OS14
KPD
MG-63
OHS
Saos-2
U-2 OS
ZK-58

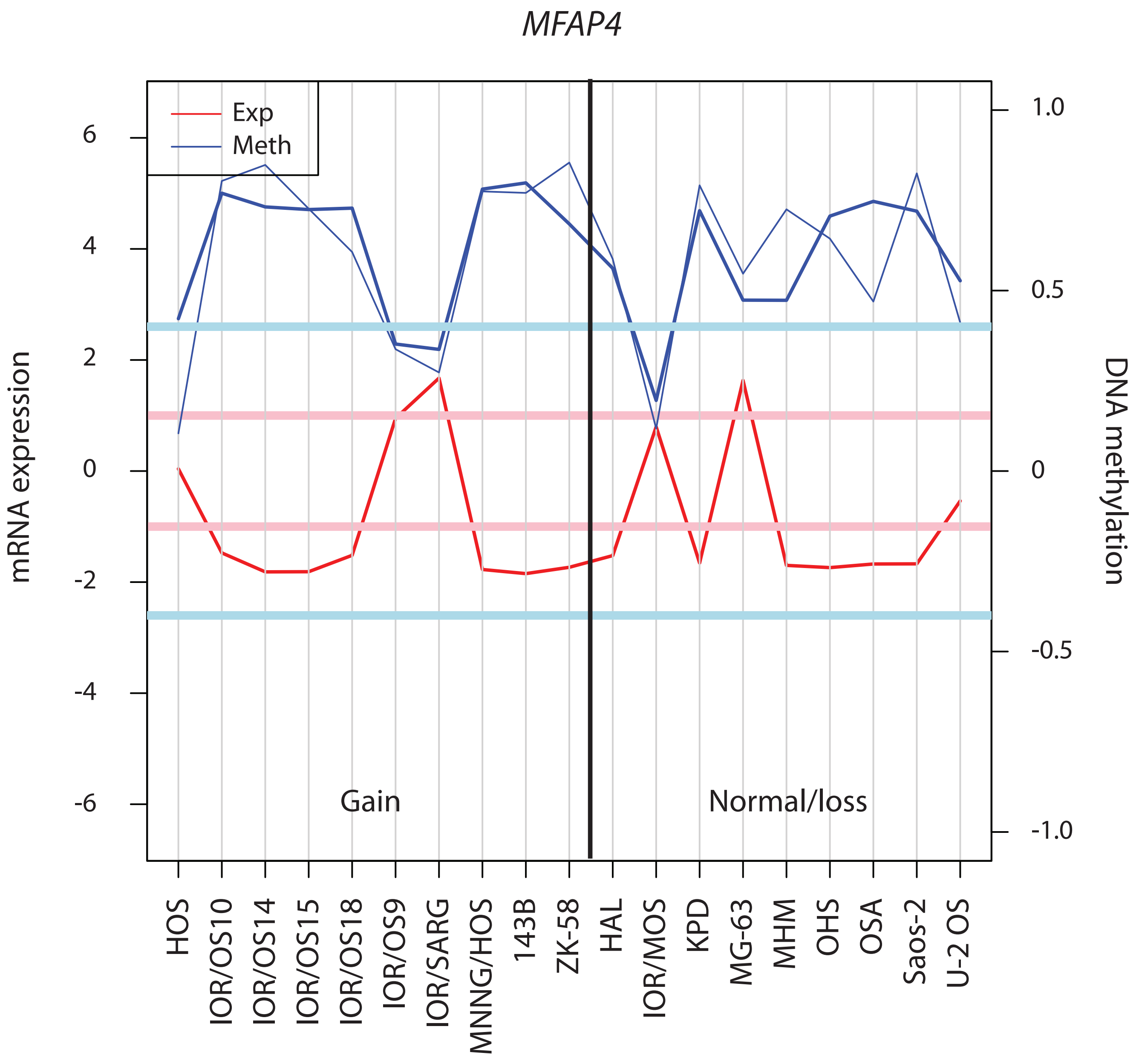

MFAP4
Exp
Meth
mRNA expression
DNA methylation
6
4
2
0
-2
-4
-6
1.0
0.5
0
-0.5
-1.0
Gain
Normal/loss
HOS
IOR/OS10
IOR/OS14
IOR/OS15
IOR/OS18
IOR/OS9
IOR/SARG
MNNG/HOS
143B
ZK-58
HAL
IOR/MOS
KPD
MG-63
MHM
OHS
OSA
Saos-2
U-2 OS

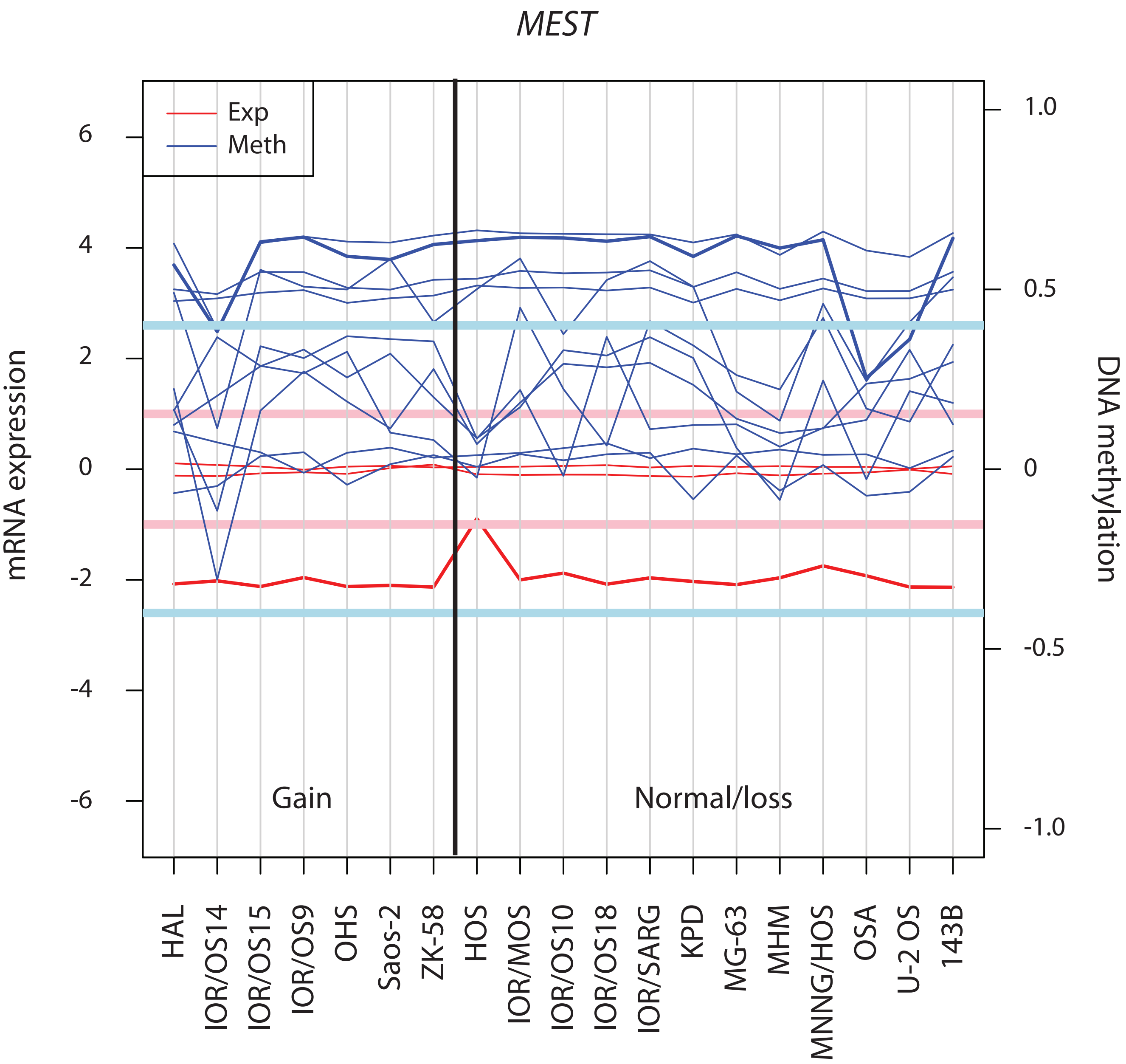

MEST
Exp
Meth
mRNA expression
DNA methylation
6
4
2
0
-2
-4
-6
1.0
0.5
0
-0.5
-1.0
Gain
Normal/loss
HAL
IOR/OS14
IOR/OS15
IOR/OS9
OHS
Saos-2
ZK-58
HOS
IOR/MOS
IOR/OS10
IOR/OS18
IOR/SARG
KPD
MG-63
MHM
MNNG/HOS
OSA
U-2 OS
143B

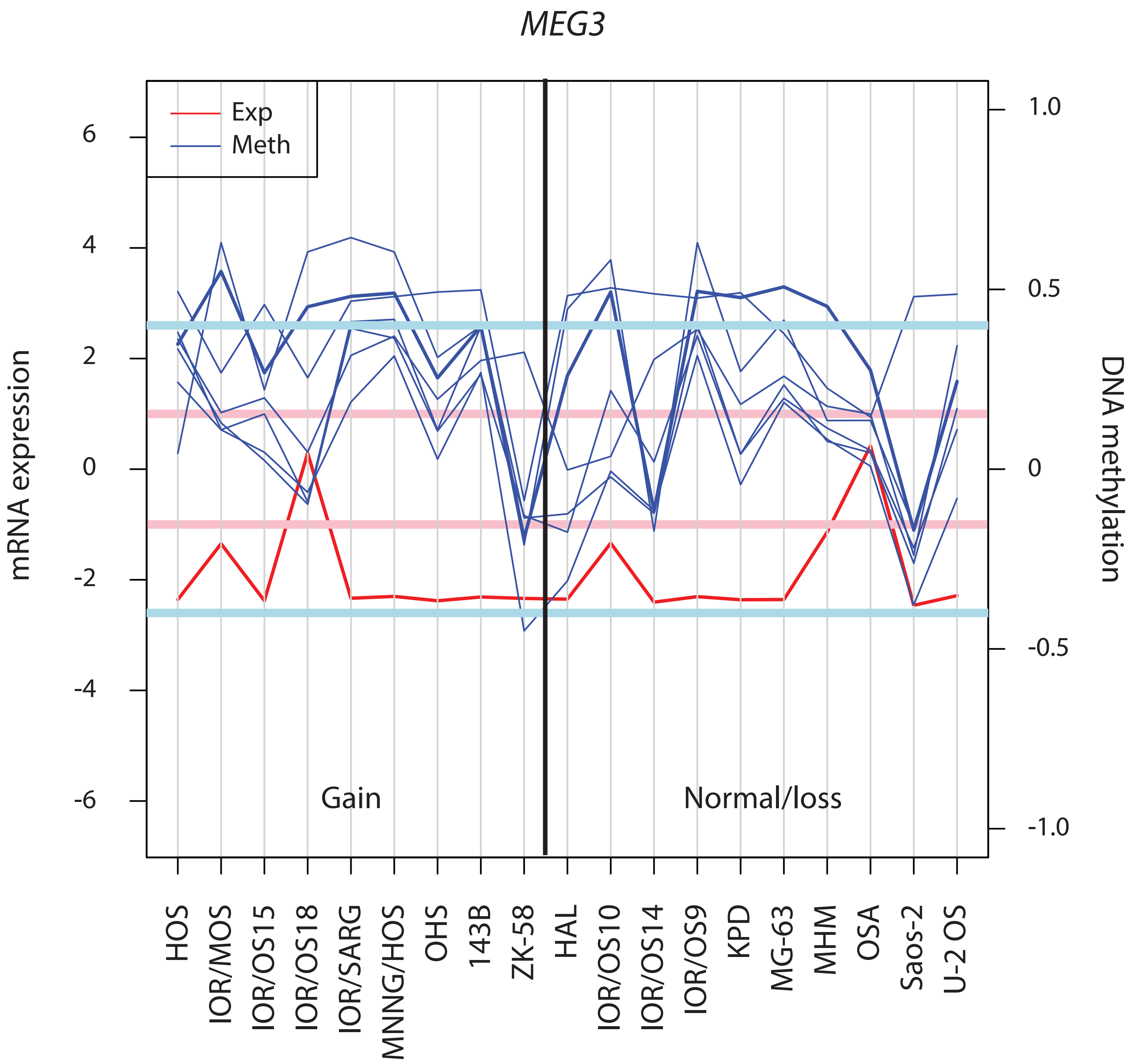

MEG3
Exp
Meth
mRNA expression
DNA methylation
6
4
2
0
-2
-4
-6
1.0
0.5
0
-0.5
-1.0
Gain
Normal/loss
HOS
IOR/MOS
IOR/OS15
IOR/OS18
IOR/SARG
MNNG/HOS
OHS
143B
ZK-58
HAL
IOR/OS10
IOR/OS14
IOR/OS9
KPD
MG-63
MHM
OSA
Saos-2
U-2 OS

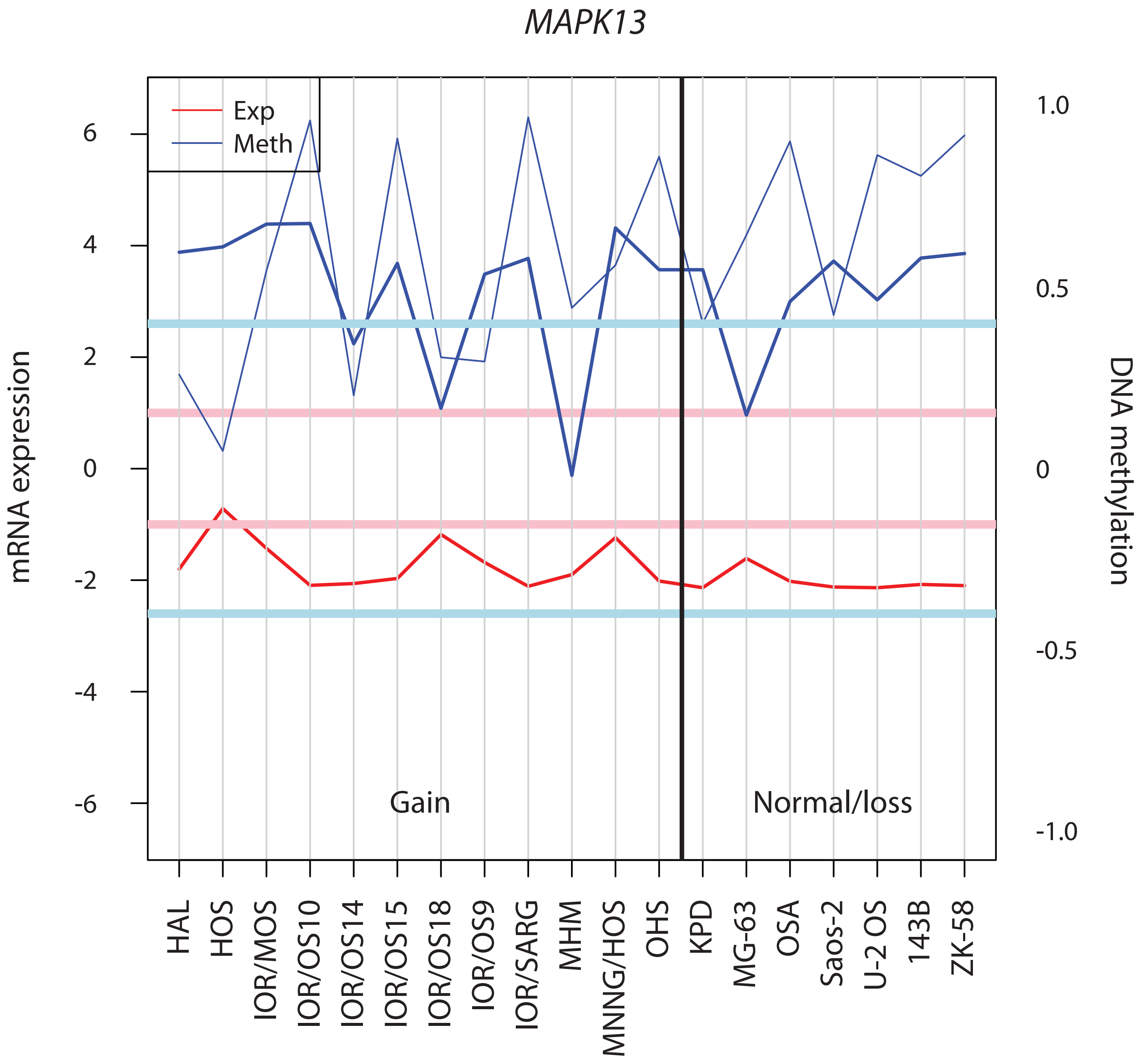

MAPK13
Exp
Meth
mRNA expression
DNA methylation
6
4
2
0
-2
-4
-6
1.0
0.5
0
-0.5
-1.0
Gain
Normal/loss
HAL
HOS
IOR/MOS
IOR/OS10
IOR/OS14
IOR/OS15
IOR/OS18
IOR/OS9
IOR/SARG
MHM
MNNG/HOS
OHS
KPD
MG-63
OSA
Saos-2
U-2 OS
143B
ZK-58

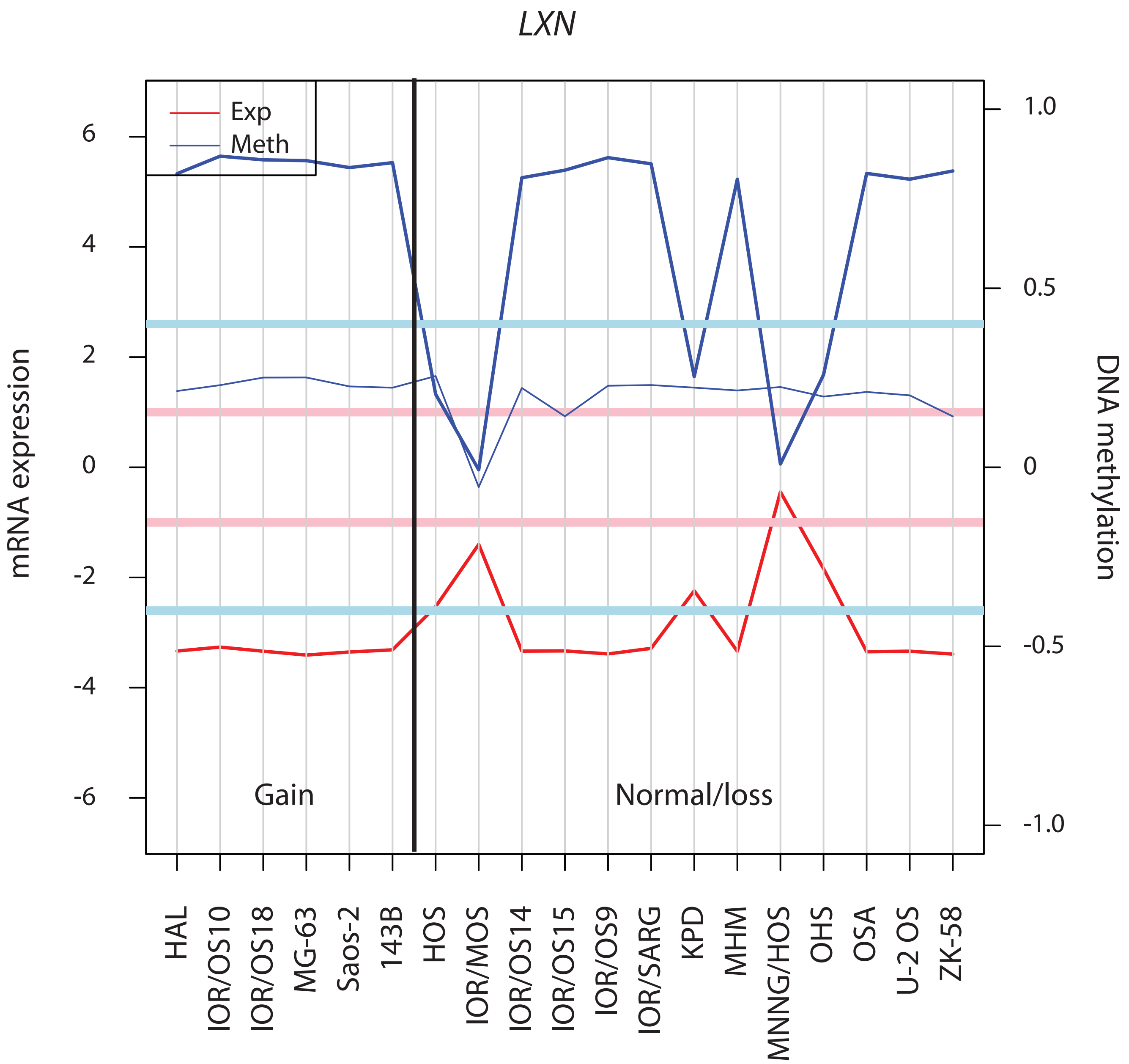

LXN
Exp
Meth
mRNA expression
DNA methylation
6
4
2
0
-2
-4
-6
1.0
0.5
0
-0.5
-1.0
Gain
Normal/loss
HAL
IOR/OS10
IOR/OS18
MG-63
Saos-2
143B
HOS
IOR/MOS
IOR/OS14
IOR/OS15
IOR/OS9
IOR/SARG
KPD
MHM
MNNG/HOS
OHS
OSA
U-2 OS
ZK-58

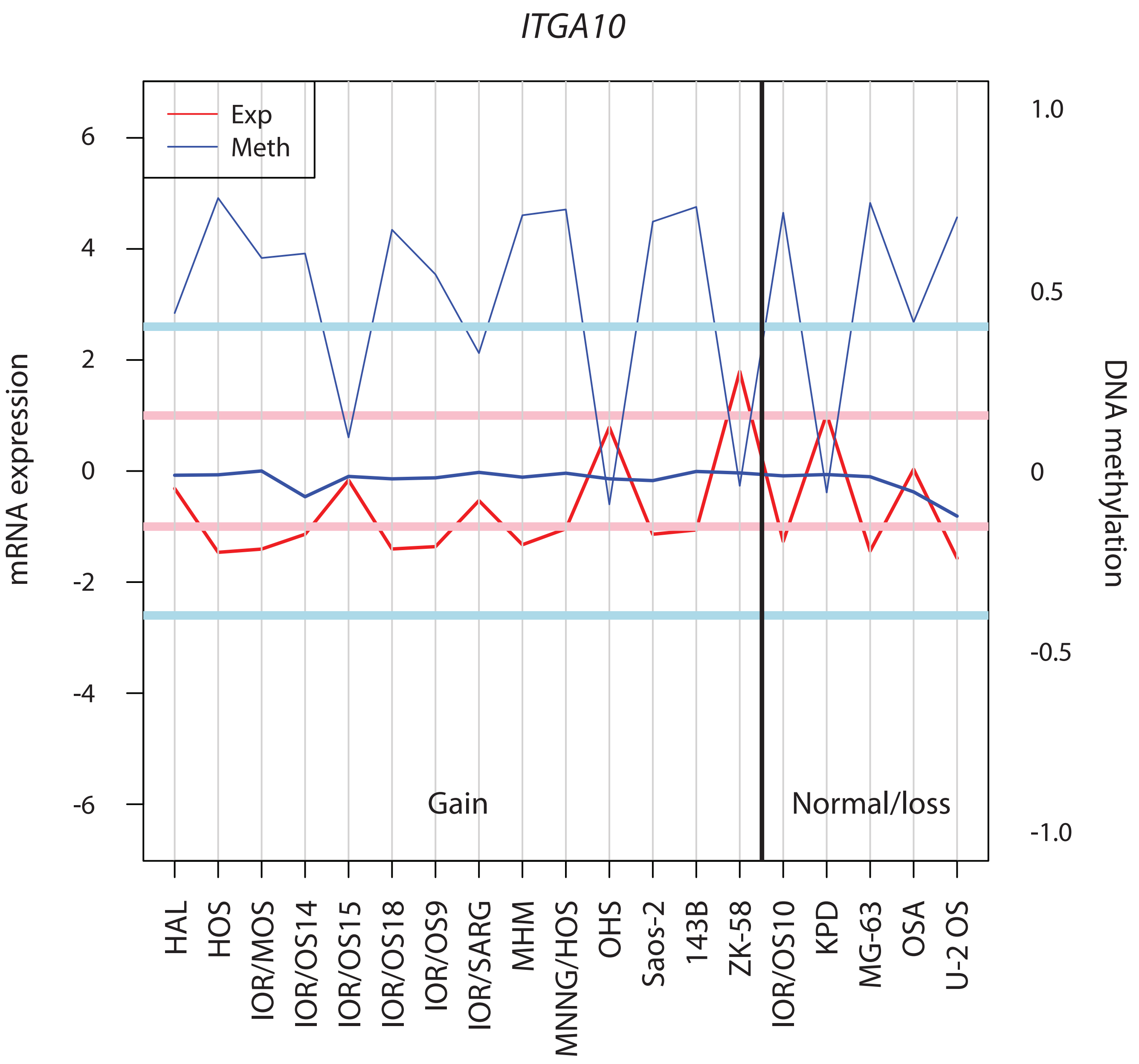

ITGA10
Exp
Meth
mRNA expression
DNA methylation
6
4
2
0
-2
-4
-6
1.0
0.5
0
-0.5
-1.0
Gain
Normal/loss
HAL
HOS
IOR/MOS
IOR/OS14
IOR/OS15
IOR/OS18
IOR/OS9
IOR/SARG
MHM
MNNG/HOS
OHS
Saos-2
143B
ZK-58
IOR/OS10
KPD
MG-63
OSA
U-2 OS

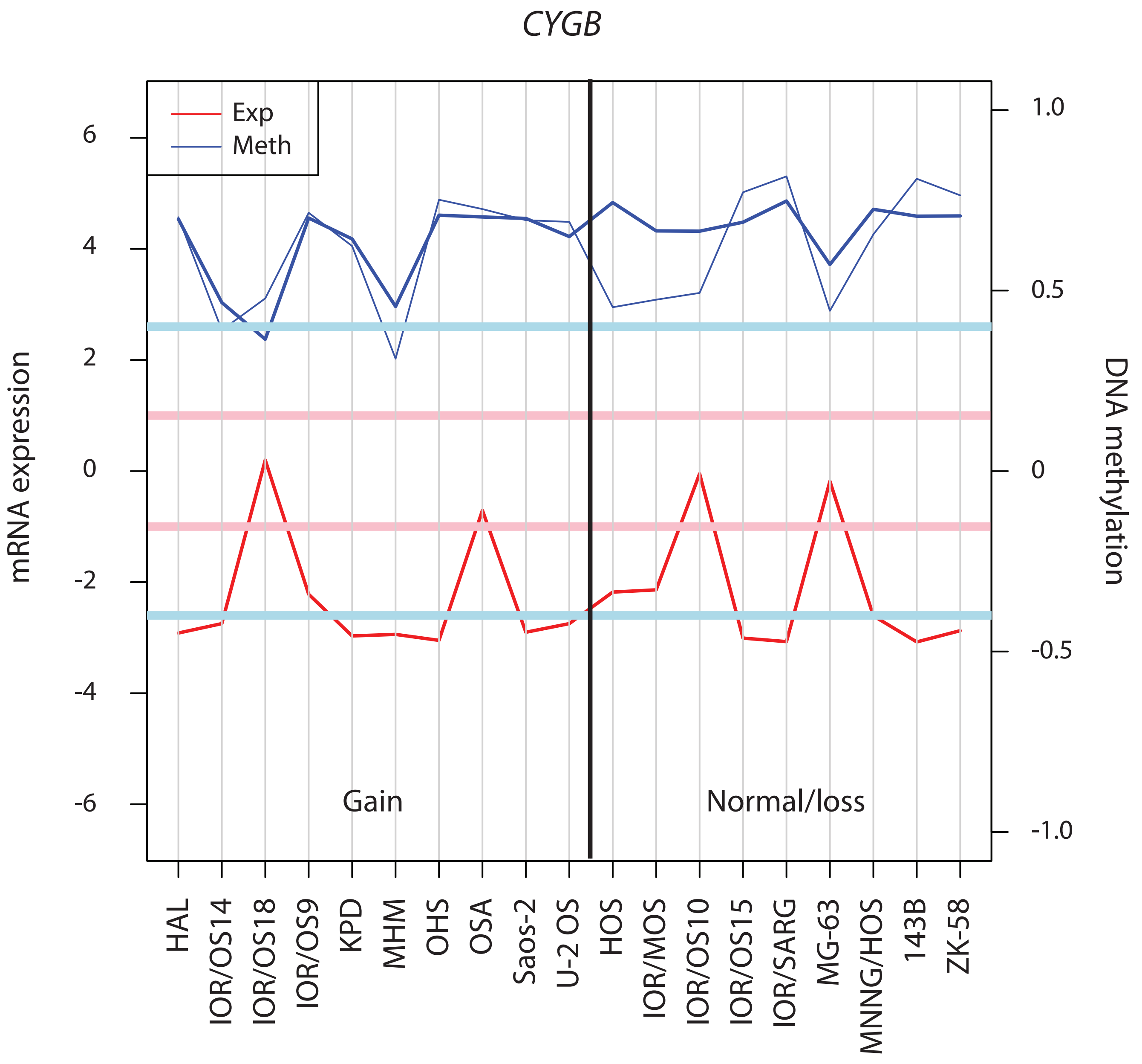

CYGB
Exp
Meth
mRNA expression
DNA methylation
6
4
2
0
-2
-4
-6
1.0
0.5
0
-0.5
-1.0
Gain
Normal/loss
HAL
IOR/OS14
IOR/OS18
IOR/OS9
KPD
MHM
OHS
OSA
Saos-2
U-2 OS
HOS
IOR/MOS
IOR/OS10
IOR/OS15
IOR/SARG
MG-63
MNNG/HOS
143B
ZK-58

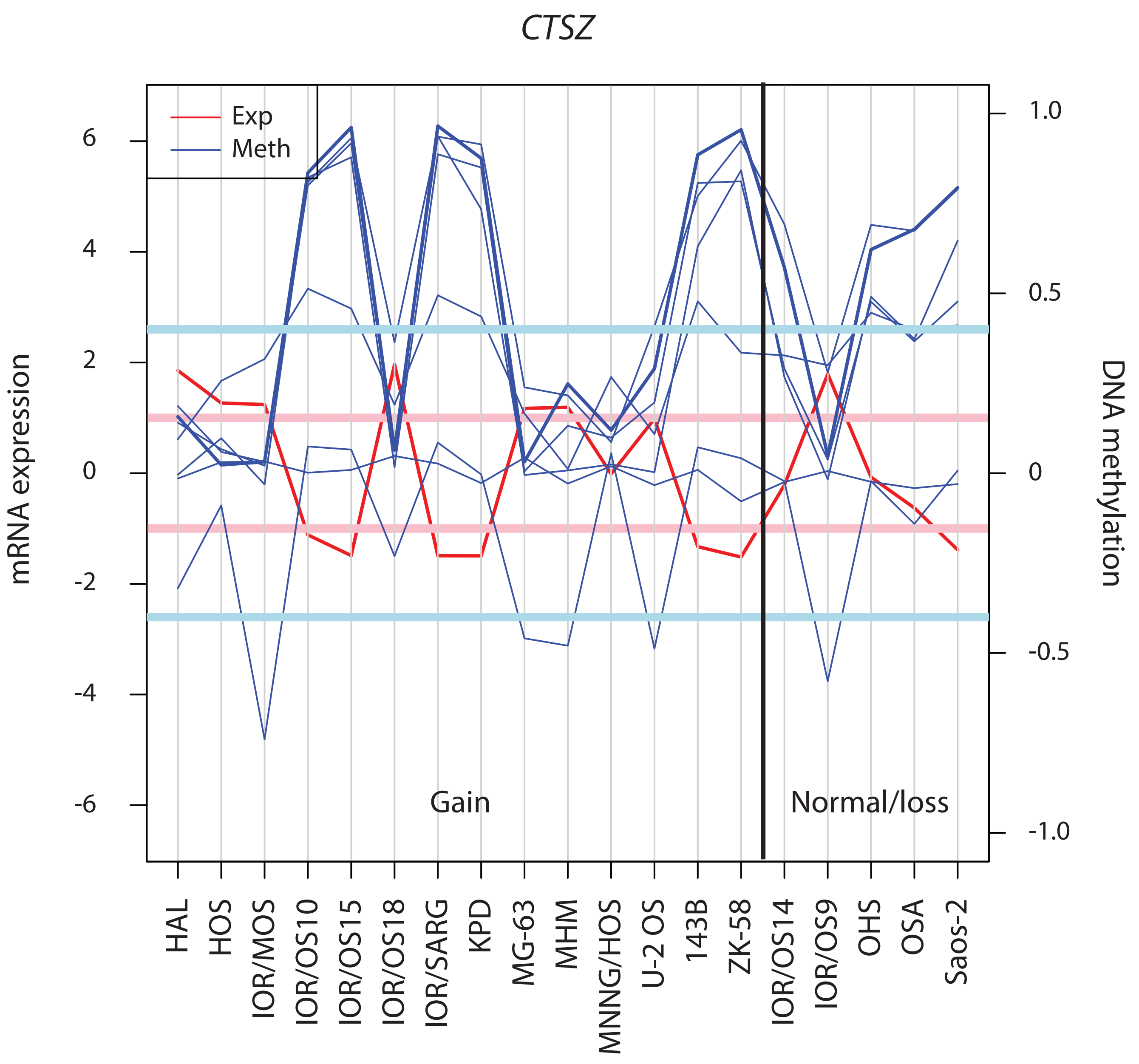
CTSZ
Exp
Meth
mRNA expression
DNA methylation
6
4
2
0
-2
-4
-6
1.0
0.5
0
-0.5
-1.0
Gain
Normal/loss
HAL
HOS
IOR/MOS
IOR/OS10
IOR/OS15
IOR/OS18
IOR/SARG
KPD
MG-63
MHM
MNNG/HOS
U-2 OS
143B
ZK-58
IOR/OS14
IOR/OS9
OHS
OSA
Saos-2

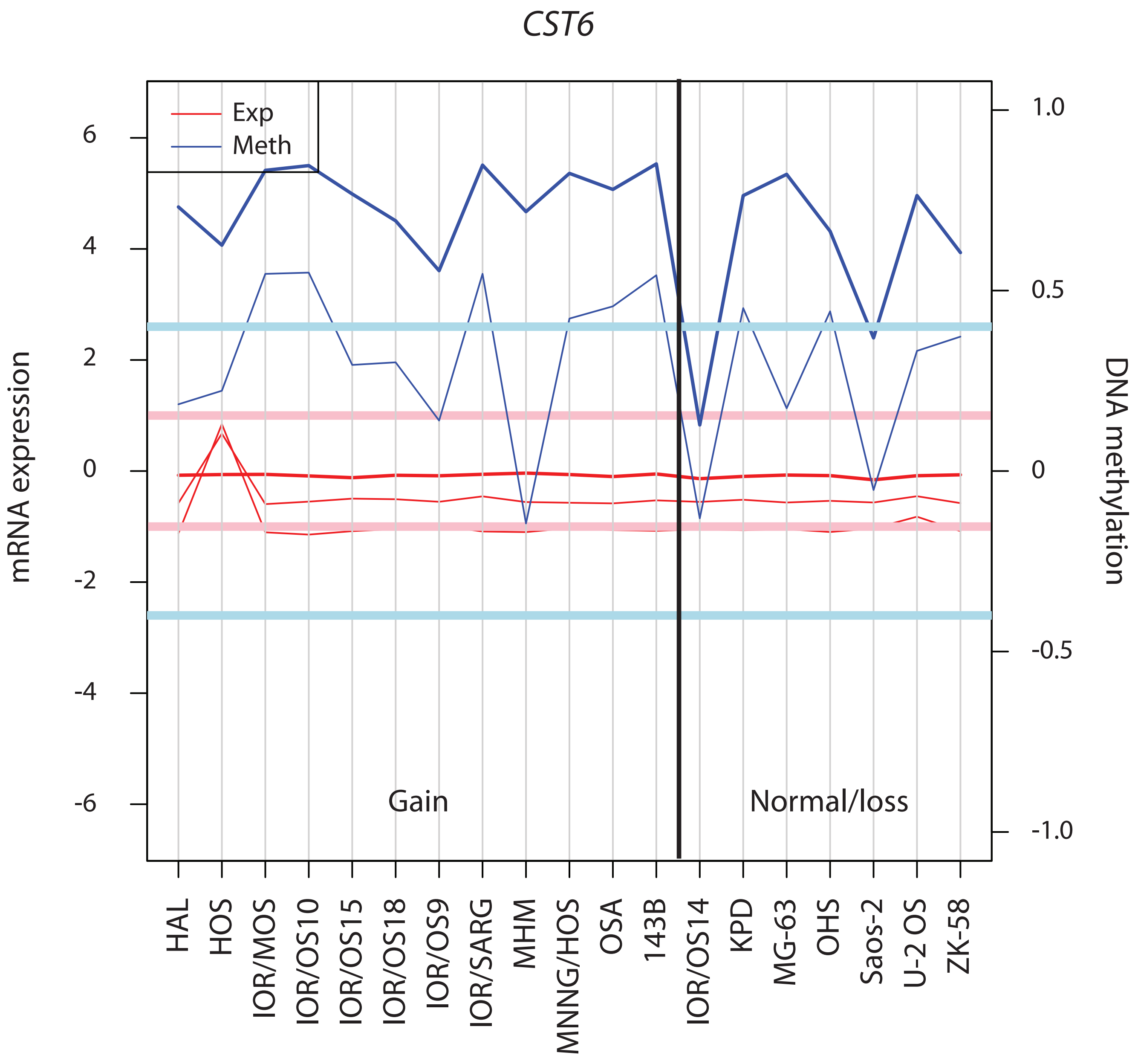

CST6
Exp
Meth
mRNA expression
DNA methylation
6
4
2
0
-2
-4
-6
1.0
0.5
0
-0.5
-1.0
Gain
Normal/loss
HAL
HOS
IOR/MOS
IOR/OS10
IOR/OS15
IOR/OS18
IOR/OS9
IOR/SARG
MHM
MNNG/HOS
OSA
143B
IOR/OS14
KPD
MG-63
OHS
Saos-2
U-2 OS
ZK-58

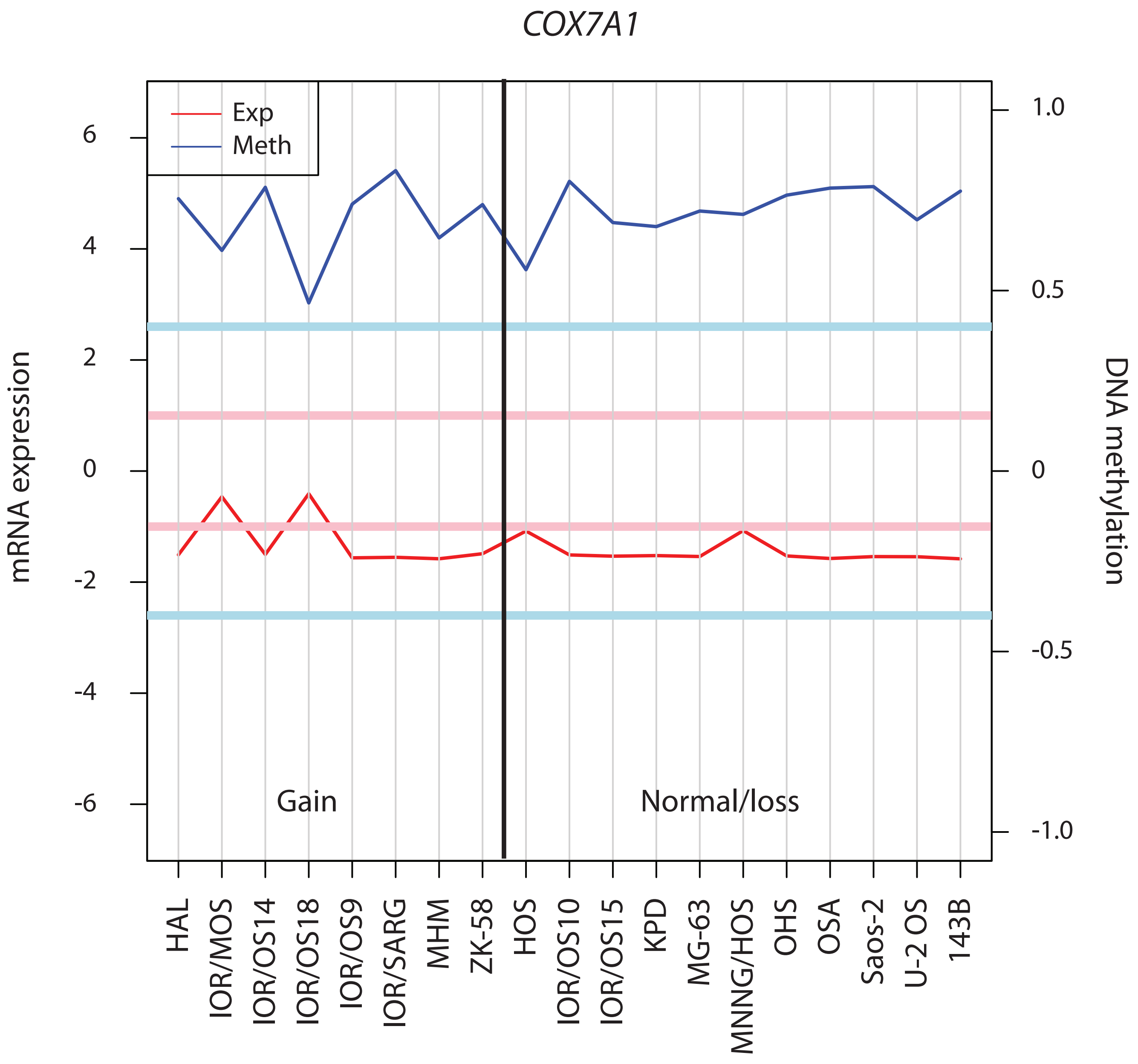

COX7A1
Exp
Meth
mRNA expression
DNA methylation
6
4
2
0
-2
-4
-6
1.0
0.5
0
-0.5
-1.0
Gain
Normal/loss
HAL
IOR/MOS
IOR/OS14
IOR/OS18
IOR/OS9
IOR/SARG
MHM
ZK-58
HOS
IOR/OS10
IOR/OS15
KPD
MG-63
MNNG/HOS
OHS
OSA
Saos-2
U-2 OS
143B

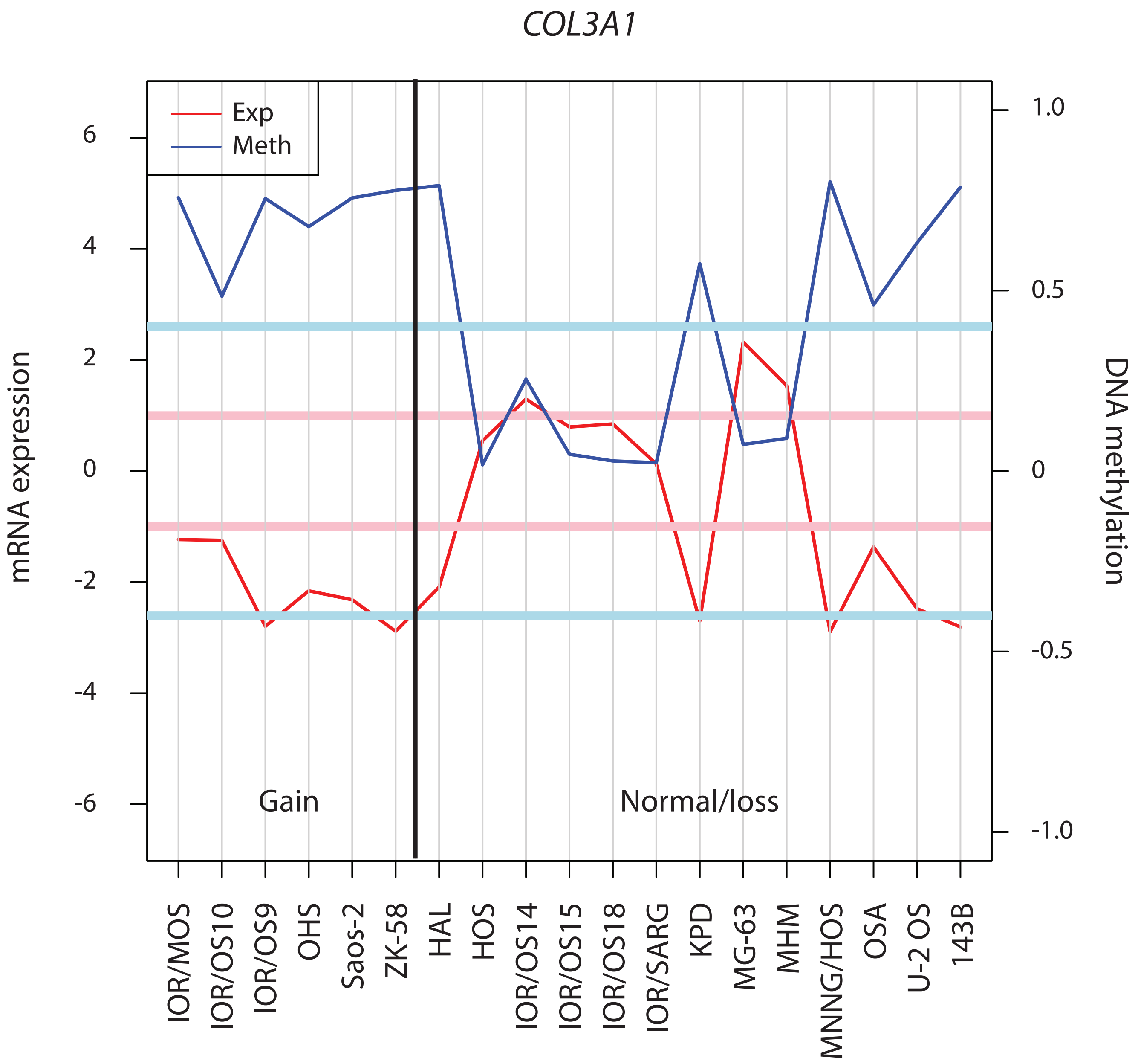

COL3A1
Exp
Meth
mRNA expression
DNA methylation
6
4
2
0
-2
-4
-6
1.0
0.5
0
-0.5
-1.0
Gain
Normal/loss
IOR/MOS
IOR/OS10
IOR/OS9
OHS
Saos-2
ZK-58
HAL
HOS
IOR/OS14
IOR/OS15
IOR/OS18
IOR/SARG
KPD
MG-63
MHM
MNNG/HOS
OSA
U-2 OS
143B
