# Supplementary material for: Integrative Analysis Reveals Relationships of Genetic and Epigenetic Alterations in Osteosarcoma
Source: PLoS One. 2012 Nov 7;7(11):e48262. doi: 10.1371/journal.pone.0048262 (PMC3492335; doi:10.1371/journal.pone.0048262)
Supplement: Table S1 — Clinical data for osteosarcoma cell lines, osteosarcoma tumour samples and normal samples. (PDF) [file pone.0048262.s009.pdf]

Table S1. Clinical data for osteosarcoma cell lines, tumour samples and normal samples (Kresse et al)

Sample	Sample type	Sample origin	Patient age (years)/sex	Subtype	Grade	Primary location	Status	ATCC number	Provider	Note
143B	Cell line	NA	13/F	Mixed	NA	NA	NA	CRL-8303	ATCC	Derived from HOS
HAL	Cell line	BM	15/M	NA	NA	NA	NA	-	The Norwegian Radium Hospital	
HOS	Cell line	NA	13/F	Fbl/epi	NA	NA	NA	CRL-1543	ATCC	
IOR/OS9	Cell line	Met	15/M	Obl	NA	NA	NA	-	Istituto Ortopedico Rizzoli	
IOR/OS10	Cell line	Prim	10/F	Fbl	NA	NA	NA	-	Istituto Ortopedico Rizzoli	
IOR/OS14	Cell line	Prim	13/M	Obl	NA	NA	NA	-	Istituto Ortopedico Rizzoli	
IOR/OS15	Cell line	Prim	12/F	Obl	NA	NA	NA	-	Istituto Ortopedico Rizzoli	
IOR/OS18	Cell line	Met	33/M	Obl	NA	NA	NA	-	Istituto Ortopedico Rizzoli	
IOR/MOS	Cell line	Prim	13/F	Obl	NA	NA	NA	-	Istituto Ortopedico Rizzoli	
IOR/SARG	Cell line	Prim	25/M	NA	NA	NA	NA	-	Istituto Ortopedico Rizzoli	
KPD	Cell line	Prim	7/M	Obl/tel	NA	NA	NA	-	The Norwegian Radium Hospital	
MG-63	Cell line	NA	14/M	Fbl	NA	NA	NA	CRL-1427	ATCC	
MHM	Cell line	Met	41/F	Fbl	NA	NA	NA	-	The Norwegian Radium Hospital	
MNNG/HOS	Cell line	NA	13/F	NA	NA	NA	NA	CRL-1547	ATCC	Derived from HOS
OHS	Cell line	Prim	14/M	Obl	NA	NA	NA	-	The Norwegian Radium Hospital	
OSA (SJSA-1)	Cell line	NA	19/M	Fbl	NA	NA	NA	CRL-2098	ATCC	
Saos-2	Cell line	NA	11/F	Epi	NA	NA	NA	HTB-85	ATCC	
U-2 OS	Cell line	NA	15/F	Epi	NA	NA	NA	HTB-96	ATCC	
ZK-58	Cell line	NA	21/M	Obl	NA	NA	NA	-	Heinrich-Heine-Universität Düsseldorf	
OS14	Tumour	Prim	14/F	Obl/fbl	4	Femur	DD	-	The Norwegian Radium Hospital	
OS29	Tumour	Prim	27/F	Obl	4	Pelvis	DD	-	The Norwegian Radium Hospital	
OS94	Tumour	Prim	33/F	Obl	4	Tibia	DD	-	The Norwegian Radium Hospital	
OS97	Tumour	Prim	12/M	Obl/cbl	4	Femur	DD	-	The Norwegian Radium Hospital	
OS99	Tumour	Prim	33/M	Obl	4	Pelvis	DD	-	The Norwegian Radium Hospital	
BONE1	Normal control	-	52/M	-	-	-	-	-	The Norwegian Radium Hospital	From renal cancer patient
BONE2	Normal control	-	20/M	-	-	-	-	-	The Norwegian Radium Hospital	From osteosarcoma patient
BONE3	Normal control	-	26/M	-	-	-	-	-	Capital Biosciences	
BONE4	Normal control	-	72/F	-	-	-	-	-	Capital Biosciences	
OB1	Normal control	-	NA	-	-	-	-	-	ScienCell Research Laboratories	
OB2	Normal control	-	NA	-	-	-	-	-	ScienCell Research Laboratories	

Abbreviations: OB, osteoblast; Prim, primary tumour; Met, metastasis; BM, bone marrow; NA, not available; M, male; F, female; Ob; osteoblastic; Fbl, fibroblastic; Tel, teleangiectatic; Epi, epithelial; Cbl, chondroblastic; DD, dead of disease
