# Supplementary material for: Integrative Analysis Reveals Relationships of Genetic and Epigenetic Alterations in Osteosarcoma
Source: PLoS One. 2012 Nov 7;7(11):e48262. doi: 10.1371/journal.pone.0048262 (PMC3492335; doi:10.1371/journal.pone.0048262)
Supplement: Table S2 — Number of genes with each type of aberration (DNA copy number, DNA methylation and mRNA expression). (PDF) [file pone.0048262.s010.pdf]

Table S2. Number of genes with each type of aberration (Kresse et al)

	DNA copy number		DNA methylation		mRNA expression	
**Sample**	**Gain**	**Loss**	**Hyper-methylation**	**Hypo-methylation**	**Over-expression**	**Under-expression**
143B	2047	295	3083	103	558	528
HAL	4135	684	1698	392	648	552
HOS	1943	454	1730	265	563	497
IOR/OS9	5475	1162	1810	233	510	342
IOR/OS10	3205	1126	2642	97	672	491
IOR/OS14	4718	1333	1052	1105	525	463
IOR/OS15	4275	1272	1992	590	578	528
IOR/OS18	5498	1569	1083	905	602	411
IOR/MOS	4510	1504	1994	194	540	435
IOR/SARG	3458	1094	2890	236	634	669
KPD	1346	3170	1831	552	607	634
MG-63	2479	599	1828	349	483	350
MHM	4773	684	1307	537	538	377
MNNG/HOS	2382	1862	2302	218	528	466
OHS	3685	917	2397	769	547	587
OSA	1201	577	1960	423	720	557
Saos-2	3102	601	1700	788	610	570
U-2 OS	2379	1974	2054	410	637	547
ZK-58	3969	931	2241	914	640	637
Total	64580	21808	37594	9080	11140	9641
